# Water quality data from estuarine variable hydrologic flow regimes during frequent drought

**DOI:** 10.1016/j.dib.2019.104178

**Published:** 2019-06-26

**Authors:** Bhanu Paudel, Paul A. Montagna, Leslie Adams

**Affiliations:** a Delaware Department of Natural Resources and Environmental Control, 100 W Water Street, Suite 10B Dover, DE 19904, USA; b Texas A&M University-Corpus Christi, Harte Research Institute for Gulf of Mexico Studies, 6300 Ocean Drive, Unit 5869 Corpus Christi, Texas 78412, USA

**Keywords:** Estuaries, Hydrologic flow, Salinity, TSS, Nutrients

## Abstract

The present article provides water quality data collected from three South Texas Estuaries (Guadalupe, Nueces and Lavaca-Colorado Estuaries) during frequent drought from 2011 to 2014. The data described here are presented in the research article “The relationship between suspended solids and nutrients with variable hydrologic flow regimes” Paudel et al., 2019. Quarterly (i.e. four times a year) surface water quality data presented here were collected from various stations lie along river-estuary mouth to oceanic salinity gradient. Followings are the water quality data provided from Texas estuaries at different river flow regimes: pH, DO, TSS, salinity, chlorophyll-a, secchi disc reading, and nutrients (dissolved nitrogen, dissolved phosphorus and dissolved silicate). Surface inflow was obtained by adding gauged, modeled and return flow.

**Table:** Specifications table

Subject area	Environmental Science
More specific subject area	Environmental Chemistry and Estuarine water quality
Type of data	Table, excel file, and SAS code in word docs.
How data was acquired	Using YSI hydro sonde (YSI Model 556 MPS) for water quality parameters; using Turner design trilogy fluorometer (Model#7200) for chlorophyll-α, and using OIA segmented flow auto-analyzer (Xylem Brand) for inorganic nutrients.
Data format	Raw and analyzed
Experimental factors	For chlorophyll-α analysis: Water samples were filtered on site and store frozen. For TSS: Samples were filtered in the laboratory and dried weights were obtained. For Nutrients: Water samples were filtered on site by 0.45 μm polycarbonate filter paper and kept frozen until analysis.
Experimental features	Data were collected from four stations in Guadalupe, five stations in Nueces, and nine stations in Lavaca-Colorado estuaries that are along river-estuary mouth to oceanic salinity gradient. Water samples were collected for chlorophyll-α, TSS and nutrients analysis.
Data source location	Water quality data were collected from three estuaries i.e. Guadalupe, Nueces, and Lavaca-Colorado in South Texas. Inflow rate data were obtained from https://waterdatafortexas.org/coastal/hydrology.
Data accessibility	The data is with this file.
Related research article	Paudel, B., P.A. Montagna, and L. Adams. 2019 “The relationship between suspended solids and nutrients with variable hydrologic flow regimes.” Regional Studies in Marine Science. https://doi.org/10.1016/j.rsma.2019.100657

**Table:** 

**Value of the data** • Data allow investigation of spatial and temporal changes in water quality parameters. • Water quality data allow investigation of changes in parameters during occurrence of drought. • Salinity data in the Nueces estuary during drought were higher near river-estuary mouth than in oceanic side, and can be compared with the estuarine data in dry regions. • Salinity data in the Nueces estuary is the evidence of “Reverse Estuary” condition, therefore data may be of interest to managers and scientists.

## 1 Data

Water quality data were provided from three micro-tidal Texas estuaries (Guadalupe, Nueces and Lavaca-Colorado estuaries) during 2011–2013 study (Table 1). Quarterly water quality data were collected along the salinity gradient (from river-estuary mouth towards the ocean inlet) from four stations in the Guadalupe, five stations in the Nueces and nine stations in the Lavaca-Colorado estuaries. Dissolved oxygen, pH, temperature, salinity, conductivity, secchi depth, dissolved inorganic nitrogen (nitrite + nitrate and ammonia), dissolved silicate, dissolved phosphate, TSS, and chlorophyll-α data were provided for surface (i.e. indicated as 0.1 m depth in Table 1) and bottom (20 cm above sediment bottom) water of the three estuaries. In the attached data file, “Date” column represents the collection date. Collection of water samples and measurements of water quality parameters from river inflow source to the estuary was categorized as “Near – near to the river source” and “Far – far to the river source” stations. Abbreviation of the estuaries’ name and water quality parameters are provided in the Table 1.

**Table 1:** Water quality measurements from 2011 to 2013 in three Texas estuaries.

DATE	Dist	STA	Est	DEPTH	TEMP	COND	SAL	DO	PH	SECCHI	PO4	SiO4	NH4	NOx	Chl	TSS
04-04-2011	Near	A	GE	0.1	24.09	25.5	15.55	7.91	8.23	0.3	0.57	64.38	2.49	2.72	21.94	74.46
04-04-2011	Near	A	GE	0.1	24.09	25.5	15.55	7.91	8.23	0.3	0.59	62.59	2.78	2.84	17.49	76.78
04-04-2011	Near	A	GE	1.2	24.04	25.74	15.7	7.68	8.22	0.3	0.67	72.94	2.49	2.59	24.27	NA
04-04-2011	Near	A	GE	1.2	24.04	25.74	15.7	7.68	8.22	0.3	0.73	73.35	2.54	2.43	27.76	NA
04-04-2011	Near	B	GE	0.1	24.19	26.83	16.47	8.21	8.29	0.3	0.6	75.01	1.37	0	28.99	63.38
04-04-2011	Near	B	GE	0.1	24.19	26.83	16.47	8.21	8.29	0.3	0.53	66.7	1.65	0.28	26.11	62.98
04-04-2011	Near	B	GE	1.7	23.41	34.18	21.48	6.75	8.08	0.3	0.67	82.47	1.77	0.35	17.92	NA
04-04-2011	Near	B	GE	1.7	23.41	34.18	21.48	6.75	8.08	0.3	0.7	72.53	1.39	0	21.44	NA
04-04-2011	Far	C	GE	0.1	23.89	36.95	23.39	7.34	8.13	0.3	0.83	59.13	1.64	0.64	9.67	60.3
04-04-2011	Far	C	GE	0.1	23.89	36.95	23.39	7.34	8.13	0.3	1.07	58.65	2.3	0.53	5.43	63.14
04-04-2011	Far	C	GE	1.8	23.61	38.51	24.52	6.88	8.13	0.3	0.8	54.45	1.65	0	6.34	NA
04-04-2011	Far	C	GE	1.8	23.61	38.51	24.52	6.88	8.13	0.3	0.83	53.63	1.95	0.26	5.74	NA
04-04-2011	Far	D	GE	0.1	23.77	35.37	22.35	7.25	8.02	0.3	0.98	53.76	2.96	0.57	5.37	80.16
04-04-2011	Far	D	GE	0.1	23.77	35.37	22.35	7.25	8.02	0.3	0.69	58.78	3.25	0.83	6.2	35.96
04-04-2011	Far	D	GE	1.4	23.41	37.16	23.57	6.72	8	0.3	0.69	48.7	3.24	0.67	4.34	NA
04-04-2011	Far	D	GE	1.4	23.41	37.16	23.57	6.72	8	0.3	0.54	48.74	3.01	0.47	5.08	NA
11-07-2011	Near	A	GE	0.1	31.52	38.22	24.1	7.46	8.22	0.4	1.29	113.04	1.02	1.36	7	47.36
11-07-2011	Near	A	GE	0.1	31.52	38.22	24.1	7.46	8.22	0.4	1.3	109.12	1.05	0.59	10.13	48.96
11-07-2011	Near	A	GE	1.4	31.5	38.21	24.1	7.42	8.22	0.4	1.3	109.69	0.93	0.46	7.04	NA
11-07-2011	Near	A	GE	1.4	31.5	38.21	24.1	7.42	8.22	0.4	1.31	100.87	0.8	0.61	10.99	NA
11-07-2011	Near	B	GE	0.1	31.21	42.34	27.02	7.03	8.26	0.6	0.94	102.77	0.95	1.77	8.43	23.36667
11-07-2011	Near	B	GE	0.1	31.21	42.34	27.02	7.03	8.26	0.6	0.94	96.22	0.41	0.38	8.73	25.1
11-07-2011	Near	B	GE	1.8	30.94	42.5	27.12	6.71	8.25	0.6	1.04	101.16	0.52	0.74	12.13	NA
11-07-2011	Near	B	GE	1.8	30.94	42.5	27.12	6.71	8.25	0.6	1.07	103.72	0.73	0.66	12.23	NA
11-07-2011	Far	C	GE	0.1	31.2	45.5	29.29	7.02	8.29	0.5	0.93	87.37	0.72	0.73	3.38	25.8
11-07-2011	Far	C	GE	0.1	31.2	45.5	29.29	7.02	8.29	0.5	0.83	86.52	0.33	0.35	15.61	32.9
11-07-2011	Far	C	GE	1.9	30.99	45.88	29.55	6.53	8.25	0.5	0.87	83.95	0.75	0.48	9.15	NA
11-07-2011	Far	C	GE	1.9	30.99	45.88	29.55	6.53	8.25	0.5	0.86	79.18	0.36	0.52	12.99	NA
11-07-2011	Far	D	GE	0.1	30.87	44.59	28.65	6.49	8.23	0.5	0.93	90.1	0.76	0.68	9.51	33.8
11-07-2011	Far	D	GE	0.1	30.87	44.59	28.65	6.49	8.23	0.5	0.95	90.66	0.48	0.54	9.63	32.73333
11-07-2011	Far	D	GE	1.5	30.63	44.63	28.68	6.14	8.24	0.5	0.96	89.36	0.84	0.46	9.05	NA
11-07-2011	Far	D	GE	1.5	30.63	44.63	28.68	6.14	8.24	0.5	0.97	89.51	0.46	0.41	9.51	NA
03-10-2011	Near	A	GE	0.1	25.22	47.3	30.76	7.35	8.07	0.4	1.78	150.17	0	1.31	8.87	66.08
03-10-2011	Near	A	GE	0.1	25.22	47.3	30.76	7.35	8.07	0.4	1.83	160.02	0	0.47	7.31	60.32
03-10-2011	Near	A	GE	1.2	25.27	47.3	30.76	7.33	8.07	0.4	1.9	149.93	0	0	7.45	NA
03-10-2011	Near	A	GE	1.2	25.27	47.3	30.76	7.33	8.07	0.4	2.66	173.29	0	0.4	12.11	NA
03-10-2011	Near	B	GE	0.1	25.32	53.34	35.19	7.1	8.02	0.5	1.7	142.13	0	0.36	10.92	55.25
03-10-2011	Near	B	GE	0.1	25.32	53.34	35.19	7.1	8.02	0.5	1.71	139.9	0	0.72	9.03	56.15
03-10-2011	Near	B	GE	1.6	25.35	53.43	35.26	7.03	8.02	0.5	1.82	115.15	0	0	10.18	NA
03-10-2011	Near	B	GE	1.6	25.35	53.43	35.26	7.03	8.02	0.5	1.74	141.92	0	0	13.1	NA
03-10-2011	Far	C	GE	0.1	25.01	55.3	36.67	7.77	8.04	0.5	1.55	134.25	0	0.41	15.45	18.24
03-10-2011	Far	C	GE	0.1	25.01	55.3	36.67	7.77	8.04	0.5	1.5	98.57	0	0	10.38	34.92
03-10-2011	Far	C	GE	1.9	24.97	55.33	36.69	7.24	8.02	0.5	1.54	130.94	0	0	17.81	NA
03-10-2011	Far	C	GE	1.9	24.97	55.33	36.69	7.24	8.02	0.5	1.61	127.4	0	0	13.73	NA
03-10-2011	Far	D	GE	0.1	24.79	55.99	37.19	6.91	8	0.5	1.4	110.35	0	0.43	10.04	36.92
03-10-2011	Far	D	GE	0.1	24.79	55.99	37.19	6.91	8	0.5	1.34	111.39	0	0.25	10.61	34.32
03-10-2011	Far	D	GE	1.5	24.79	55.99	37.19	7.75	8.01	0.5	1.29	116.25	0	0	8.07	NA
03-10-2011	Far	D	GE	1.5	24.79	55.99	37.19	7.75	8.01	0.5	1.31	114.99	0	0	8.68	NA
04-01-2012	Near	A	GE	0.1	14.83	38.9	24.3	12.8	8.59	0.7	0.35	1.16	0.28	11.5	12.51	16.44
04-01-2012	Near	A	GE	0.1	14.83	38.9	24.3	12.8	8.59	0.7	0.35	1.2	0.37	11.5	13.73	18
04-01-2012	Near	A	GE	1.1	14.84		26.06	14.09	8.56	0.7	0.39	1.55	1.17	8.51	15.75	NA
04-01-2012	Near	A	GE	1.1	14.84		26.06	14.09	8.56	0.7	0.59	1.36	1.29	8.71	14.29	NA
04-01-2012	Near	B	GE	0.1	14.7	42.7	27	11.18	8.51	0.7	0.14	1.25	0.29	1.09	8.88	18.12
04-01-2012	Near	B	GE	0.1	14.7	42.7	27	11.18	8.51	0.7	0.16	1.15	0.34	0.69	9.91	16.2
04-01-2012	Near	B	GE	1.5	13.42		28.73	10.33	8.4	0.7	0.22	1.3	0.29	0.29	9.5	NA
04-01-2012	Near	B	GE	1.5	13.42		28.73	10.33	8.4	0.7	0.25	1.46	0.61	0.41	5.04	NA
04-01-2012	Far	C	GE	0.1	13.47	47.6	30.7	9.48	8.31	0.7	0.31	1.05	0.31	0.35	4.06	20.36
04-01-2012	Far	C	GE	0.1	13.47	47.6	30.7	9.48	8.31	0.7	0.32	1.24	0.43	0	2.7	18.32
04-01-2012	Far	C	GE	1.7	13.39		31.64	9.94	8.29	0.7	0.38	1.44	0.68	0	5.3	NA
04-01-2012	Far	C	GE	1.7	13.39		31.64	9.94	8.29	0.7	0.43	1.18	0.99	0	3.38	NA
04-01-2012	Far	D	GE	0.1	14.49	49	31.7	8.94	8.07	1.6	0.25	2.97	0.37	0.48	0.48	23.65714
04-01-2012	Far	D	GE	0.1	14.49	49	31.7	8.94	8.07	1.6	0.24	2.98	0	0	0.77	17.25714
04-01-2012	Far	D	GE	1.3	13.38		31.5	9.2	8.11	1.6	0.27	3.09	0.49	0	1.43	NA
04-01-2012	Far	D	GE	1.3	13.38		31.5	9.2	8.11	1.6	0.3	3.31	1.24	0	0.87	NA
02-04-2012	Near	A	GE	0.1	25.27	3.67	1.93	7.69	8.25	0.5	0.57	223.17	7.02	56.5	7.67	281.6
02-04-2012	Near	A	GE	0.1	25.27	3.67	1.93	7.69	8.25	0.5	0.59	237.81	7.13	51.1	11.22	277
02-04-2012	Near	A	GE	1.3	25.26	3.66	1.92	7.6	8.22	0.5	0.67	269.08	7.24	52.8	8.59	NA
02-04-2012	Near	A	GE	1.3	25.26	3.66	1.92	7.6	8.22	0.5	0.73	238.99	7.29	51.7	9.7	NA
02-04-2012	Near	B	GE	0.1	25.44	18.48	10.93	7.86	8.36	0.1	0.6	138.77	2.64	4.01	26.47	79.33333
02-04-2012	Near	B	GE	0.1	25.44	18.48	10.93	7.86	8.36	0.1	0.53	147.07	2.14	4.8	20.98	93.6
02-04-2012	Near	B	GE	1.8	25.48	18.82	11.06	7.55	8.35	0.1	0.67	138.1	2.03	3.94	31.76	NA
02-04-2012	Near	B	GE	1.8	25.48	18.82	11.06	7.55	8.35	0.1	0.7	142.19	1.89	4.15	24.88	NA
02-04-2012	Far	C	GE	0.1	25.43	31.75	19.76	7.3	8.18	0.2	0.83	60.59	0.98	0.94	10.8	61.73333
02-04-2012	Far	C	GE	0.1	25.43	31.75	19.76	7.3	8.18	0.2	1.07	61.44	0.65	0	8.13	71.06667
02-04-2012	Far	C	GE	1.9	25.34	32.12	20.02	7.11	8.14	0.2	0.8	60.86	0.85	0	15.16	NA
02-04-2012	Far	C	GE	1.9	25.34	32.12	20.02	7.11	8.14	0.2	0.83	59.79	0.83	0	8.03	NA
02-04-2012	Far	D	GE	0.1	25.22	30.97	19.23	6.97	8.07	0.1	0.98	83.34	1.67	0.26	9.76	122.4
02-04-2012	Far	D	GE	0.1	25.22	30.97	19.23	6.97	8.07	0.1	0.69	82.21	1.52	0.47	11.46	176.8
02-04-2012	Far	D	GE	1.5	25.24	30.96	19.22	6.96	8.06	0.1	0.69	81.6	1.7	0.41	6.37	NA
02-04-2012	Far	D	GE	1.5	25.24	30.96	19.22	6.96	8.06	0.1	0.54	84.52	1.64	0	7.42	NA
10-07-2012	Near	A	GE	0.1	28.61	15.51	9	8.71	8.47	0.4	2.73	183.11	0.98	0.25	22.38	38.45
10-07-2012	Near	A	GE	0.1	28.61	15.51	9	8.71	8.47	0.4	2.78	183	0.86	0.74	16.04	28.65
10-07-2012	Near	A	GE	1.1	28.26	21.87	13.13	6.16	8.29	0.4	2.59	161.69	0.75	0	19.63	NA
10-07-2012	Near	A	GE	1.1	28.26	21.87	13.13	6.16	8.29	0.4	2.56	161.05	1.03	0.46	18.76	NA
10-07-2012	Near	B	GE	0.1	28.84	23.79	14.39	7.95	8.52	0.5	1.73	161.65	0.76	2.2	18.33	21.33333
10-07-2012	Near	B	GE	0.1	28.84	23.79	14.39	7.95	8.52	0.5	1.73	158.64	0.85	0.75	17.7	51.53333
10-07-2012	Near	B	GE	1.4	28.76	28.16	17.26	5.97	8.39	0.5	1.62	137.75	0.73	0	18.54	NA
10-07-2012	Near	B	GE	1.4	28.76	28.16	17.26	5.97	8.39	0.5	1.64	152.93	1.02	0.44	14.16	NA
10-07-2012	Far	C	GE	0.1	28.76	32.2	20	7.4	8.43	0.6	1.04	121.68	0.75	0.55	16.29	21.25714
10-07-2012	Far	C	GE	0.1	28.76	32.2	20	7.4	8.43	0.6	1.04	112.36	0.6	0.48	12.64	20.74286
10-07-2012	Far	C	GE	1.6	28.89	38.89	24.67	5.58	8.27	0.6	0.85	107.3	0.69	0	13.65	NA
10-07-2012	Far	C	GE	1.6	28.89	38.89	24.67	5.58	8.27	0.6	0.88	107.23	0.74	0	13.43	NA
10-07-2012	Far	D	GE	0.1	28.36	31.65	19.63	6.89	8.37	0.4	1.23	118.99	0.66	0.52	0.42	30.4
10-07-2012	Far	D	GE	0.1	28.36	31.65	19.63	6.89	8.37	0.4	1.22	119.52	0.74	0.65	12.21	40.26667
10-07-2012	Far	D	GE	1.3	28.24	32	19.88	6.29	8.33	0.4	1.19	119.66	0.74	0	11.12	NA
10-07-2012	Far	D	GE	1.3	28.24	32	19.88	6.29	8.33	0.4	1.21	116.49	0.96	0	11.48	NA
02-10-2012	Near	A	GE	0.1	24.99	23.43	14.18	9.96	8.47	0.4	2.71	194.09	1.92	0	23.28	25.96
02-10-2012	Near	A	GE	0.1	24.99	23.43	14.18	9.96	8.47	0.4	2.96	196.21	2.22	0	25.56	36.28
02-10-2012	Near	A	GE	1	24.08	25.06	15.6	9.15	8.44	0.4	3.3	257.14	2.49	0.35	26.25	NA
02-10-2012	Near	A	GE	1	24.08	25.06	15.6	9.15	8.44	0.4	3.09	187.8	1.77	0	27.79	NA
02-10-2012	Near	B	GE	0.1	24.44	28.44	17.53	8.95	8.43	0.4	3.09	208.56	1.97	0.34	34.77	42.68
02-10-2012	Near	B	GE	0.1	24.44	28.44	17.53	8.95	8.43	0.4	3.08	202.15	1.73	0.43	32.71	40.76
02-10-2012	Near	B	GE	1.4	23.69	29.6	18.25	7.86	8.37	0.4	3.18	256.33	1.47	0	35.02	40.76
02-10-2012	Near	B	GE	1.4	23.69	29.6	18.25	7.86	8.37	0.4	2.62	147.47	0.52	0	28.25	40.76
02-10-2012	Far	C	GE	0.1	24.57	44.4	28.68	7.58	8.26	0.5	1.81	118.26	1.27	0.48	15.29	26.52
02-10-2012	Far	C	GE	0.1	24.57	44.4	28.68	7.58	8.26	0.5	1.89	137	0.53	0.41	17.74	29.36
02-10-2012	Far	C	GE	1.6	24.17	44.65	28.86	7.02	8.24	0.5	2.13	137.57	1.24	0	19.54	29.36
02-10-2012	Far	C	GE	1.6	24.17	44.65	28.86	7.02	8.24	0.5	2.32	125.67	0.74	0	12.6	29.36
02-10-2012	Far	D	GE	0.1	24.13	29.97	18.51	8.56	8.37	0.6	2.19	149.19	2.96	3.44	11.19	28.73333
02-10-2012	Far	D	GE	0.1	24.13	29.97	18.51	8.56	8.37	0.6	2.53	159.22	2.1	0	15.89	27.46667
02-10-2012	Far	D	GE	1.3	23.58	33.31	20.93	7.54	8.29	0.6	2.43	166.18	2.06	0.31	17.96	NA
02-10-2012	Far	D	GE	1.3	23.58	33.31	20.93	7.54	8.29	0.6	2.89	163.91	2.93	0	22.3	NA
11-01-2013	Near	A	GE	0.1	14.79	32.96	20.68	11.55	8.5	0.8	1.39	46.35	0.5	23.1	15.3	20.8
11-01-2013	Near	A	GE	0.1	14.79	32.96	20.68	11.55	8.5	0.8	1.39	46.35	0.5	23.1	18.81	15.2
11-01-2013	Near	A	GE	1.2	14.3	38.25	24.36	13.07	8.46	0.8	1.47	36.19	0.9	29.61	13.27	22.43333
11-01-2013	Near	A	GE	1.2	14.3	38.25	24.36	13.07	8.46	0.8	0.65	36.15	0.47	6.79	16.19	22.43333
11-01-2013	Near	B	GE	0.1	14.1	38.19	24.29	10.52	8.4	1.3	0.5	38.31	0.35	3.86	10.44	11.22857
11-01-2013	Near	B	GE	0.1	14.1	38.19	24.29	10.52	8.4	1.3	0.53	37.58	0.61	3.23	8.3	11.25714
11-01-2013	Near	B	GE	1.7	13.85	43.01	27.67	11.24	8.35	1.3	0.62	33.41	0.36	0.25	5.13	21.66667
11-01-2013	Near	B	GE	1.7	13.85	43.01	27.67	11.24	8.35	1.3	0.63	33.15	0.39	0	4.43	21.66667
11-01-2013	Far	C	GE	0.1	13.67	44.02	28.37	9.15	8.21	1.3	0.56	36.13	0.62	1.12	1.96	8.8
11-01-2013	Far	C	GE	0.1	13.67	44.02	28.37	9.15	8.21	1.3	0.57	36.43	0.48	0.83	2.16	10.14286
11-01-2013	Far	C	GE	1.9	13.55	46.26	29.9	9.39	8.18	1.3	0.56	31.64	0.37	0	4.75	26.13333
11-01-2013	Far	C	GE	1.9	13.55	46.26	29.9	9.39	8.18	1.3	0.57	31.33	0.33	0	4.42	26.13333
11-01-2013	Far	D	GE	0.1	13.82	43.02	27.7	9.71	8.27	1.6	0.39	29.38	0.87	0.28	5.55	15.51429
11-01-2013	Far	D	GE	0.1	13.82	43.02	27.7	9.71	8.27	1.6	0.39	29.27	0.34	0.61	5.19	9.657143
11-01-2013	Far	D	GE	1.5	14	49.04	32.07	8.16	8.12	1.6	0.48	27.24	1.01	0	3.49	13.36667
11-01-2013	Far	D	GE	1.5	14	49.04	32.07	8.16	8.12	1.6	0.48	25.75	0.78	0	3.55	13.36667
15-04-2013	Near	A	GE	0.1	23.1	34.63	21.79	9.4	8.41	0.3	0.18	33.27	0.95	1.04	37.09	132.5
15-04-2013	Near	A	GE	0.1	23.1	34.63	21.79	9.4	8.41	0.3	0.18		1.01	1.01	40.47	134.85
15-04-2013	Near	A	GE	1.5	23.1	34.63	21.79	9.4	8.41	0.3	0.19	30.62	0.93	1.07	40.47	182.15
15-04-2013	Near	A	GE	1.5	23.51	34.95	22.01	8.11	8.39	0.3	0.19	29.55	1.89	0.28	38.36	181.15
15-04-2013	Near	B	GE	0.1	23.38	41.98	26.97	7.12	8.27	0.4	0.15	22.13	2.72	1.14	13.97	55.95
15-04-2013	Near	B	GE	0.1	23.38	41.98	26.97	7.12	8.27	0.4	0.2	21.14	6.44		12.27	56.15
15-04-2013	Near	B	GE	2	23.1	42.21	27.12	7.1	8.26	0.4	0.16	22.67	6.06		13.53	140.05
15-04-2013	Near	B	GE	2	23.1	42.21	27.12	7.1	8.26	0.4	0.16	20.87	1.71		10.38	116.75
15-04-2013	Far	C	GE	0.1	23.01	44.63	28.87	7.49	8.14	0.4	0.42	28.28	1.24	3.17	7.36	50.05
15-04-2013	Far	C	GE	0.1	23.01	44.63	28.87	7.49	8.14	0.4	0.45	28.7	1.23	1.02	5.18	48.4
15-04-2013	Far	C	GE	2.1	22.57	45.1	29.24	7.04	8.11	0.4	0.44	28.26	0.99	0.26	11.99	324.0667
15-04-2013	Far	C	GE	2.1	22.57	45.1	29.24	7.04	8.11	0.4	0.46	31.9	7.68		7.01	308
15-04-2013	Far	D	GE	0.1	23.09	49.65	32.53	7.16	7.96	0.5	0.26	17.59	0.95	3.48	4.19	38.35
15-04-2013	Far	D	GE	0.1	23.09	49.65	32.53	7.16	7.96	0.5	0.26	17.36	1.24		2.34	41.35
15-04-2013	Far	D	GE	1.6	22.64	50.41	33.09	6.69	7.96	0.5	0.33	20.22	1.52	0.33	6.4	53.7
15-04-2013	Far	D	GE	1.6	22.64	50.41	33.09	6.69	7.96	0.5	0.33	20.29	2.12		4.58	54.15
01-10-2013	Near	A	GE	0.1	28.77	39.32	24.96	6.67	8.25	0.4			3.62	2.6	26.16	98.5
01-10-2013	Near	A	GE	0.1	28.77	39.32	24.96	6.67	8.25	0.4	1.5	134.25	3.61	2.56	34.55	98.93333
01-10-2013	Near	A	GE	1.4	28.77	39.32	24.96	6.67	8.25	0.4	1.56	146.47	3.91	1.75	36.88	111.6
01-10-2013	Near	A	GE	1.4	28.81	39.35	24.97	6.77	8.26	0.4	1.48	142.68	4.3	2.94	28.55	108.64
01-10-2013	Near	B	GE	0.1	28.9	43.32	28.17	6.52	8.13	0.4	1.79	127.48	1.03	0.72	17.62	101.1
01-10-2013	Near	B	GE	0.1	28.9	43.32	28.17	6.52	8.13	0.4	1.74	127.11	2.24	0.42	20.64	100.9667
01-10-2013	Near	B	GE	1.9	28.92	44.02	28.29	6.43	8.14	0.4	1.75	119.97	1.4	0.33	14.63	110.44
01-10-2013	Near	B	GE	1.9	28.92	44.02	28.29	6.43	8.14	0.4	1.78	131.9	1.27	0.99	14.19	111.12
01-10-2013	Far	C	GE	0.1	28.98	46.94	30.41	6.91	8	0.5	0.92	93.91	1	1.84	16.94	44.93333
01-10-2013	Far	C	GE	0.1	28.98	46.94	30.41	6.91	8	0.5	0.92	86.16	1.75	1.81	19.38	47.2
01-10-2013	Far	C	GE	1.8	28.98	47.33	30.68	6.87	8.03	0.5	0.94	86.52	1.6	1.2	15.13	107.1333
01-10-2013	Far	C	GE	1.8	28.98	47.33	30.68	6.87	8.03	0.5	0.94	87.24	1.82	1.21	14.63	104.2333
01-10-2013	Far	D	GE	0.1	28.76	47.48	30.85	6.17	7.64	0.7	0.53	61.65	1.9	1.7	5.77	17.37143
01-10-2013	Far	D	GE	0.1	28.76	47.48	30.85	6.17	7.64	0.7	0.54	61.36	1.34	0.51	5.86	22.08571
01-10-2013	Far	D	GE	1.6	28.77	47.86	31.08	6.15	7.73	0.7	0.6	65.12	2.17	0.84	6.61	17.74286
01-10-2013	Far	D	GE	1.6	28.77	47.86	31.08	6.15	7.73	0.7	0.56	63.88	2.46	0.93	7.17	17.51429
05-04-2011	Far	15	LC	0.1	20.01	48.33	31.58	8.19	8.07	0.3	1.47	37.29	4.82	2.44	6.72	NA
05-04-2011	Far	15	LC	0.1	20.01	48.33	31.58	8.19	8.07	0.3	1.42	36.1	5.64	3.01	6.2	NA
05-04-2011	Far	15	LC	0.6	20.06	48.33	31.58	8.13	8.07	0.3	1.52	39.07	5.06	2.37	8.5	NA
05-04-2011	Far	15	LC	0.6	20.06	48.33	31.58	8.13	8.07	0.3	1.29	28.94	5.79	2.51	8.37	NA
05-04-2011	Far	8	LC	0.1	21.31	50.29	33	7.4	8.26	0.8	0.65	16.46	0.29	0	3.56	NA
05-04-2011	Far	8	LC	0.1	21.31	50.29	33	7.4	8.26	0.8	0.6	15.73	0	0	3.05	NA
05-04-2011	Far	8	LC	2.4	21.03	50.21	32.95	7.3	8.24	0.8	0.64	16.44	0.25	0.98	3.53	NA
05-04-2011	Far	8	LC	2.4	21.03	50.21	32.95	7.3	8.24	0.8	0.64	16.55	0	0	4.13	NA
05-04-2011	Near	A	LC	0.1	21.45	45.24	29.36	8.17	8.13	0.7	0.44	39.4	0.47	0.52	3.62	16.04
05-04-2011	Near	A	LC	0.1	21.45	45.24	29.36	8.17	8.13	0.7	0.39	34.84	0.7	0	4.63	25.9
05-04-2011	Near	A	LC	1.2	21.44	45.4	29.44	8.3	8.14	0.7	0.4	36.39	0.52	0	3.93	NA
05-04-2011	Near	A	LC	1.2	21.44	45.4	29.44	8.3	8.14	0.7	0.41	37.29	1.18	0	5.13	NA
05-04-2011	Near	B	LC	0.1	21.79	46.06	29.91	8.14	8.16	0.7	0.49	33.9	0.59	0	6.83	26.5
05-04-2011	Near	B	LC	0.1	21.79	46.06	29.91	8.14	8.16	0.7	0.48	34.11	0.79	0	4.22	30.78
05-04-2011	Near	B	LC	1.7	21.84	46.14	29.97	8.07	8.16	0.7	0.47	33.52	0.44	0	6.05	NA
05-04-2011	Near	B	LC	1.7	21.84	46.14	29.97	8.07	8.16	0.7	0.46	31.84	0.55	0	4.7	NA
05-04-2011	Far	C	LC	0.1	20.74	48.32	31.58	7.38	8.1	0.5	0.35	15.77	0.99	0.47	3.73	30.02
05-04-2011	Far	C	LC	0.1	20.74	48.32	31.58	7.38	8.1	0.5	0.35	15.71	0.92	0	4	36.1
05-04-2011	Far	C	LC	2.6	20.72	48.54	31.76	7.25	8.12	0.5	0.36	15.4	1.11	0	4.52	NA
05-04-2011	Far	C	LC	2.6	20.72	48.54	31.76	7.25	8.12	0.5	0.36	15.32	0.94	0	3.27	NA
05-04-2011	Far	D	LC	0.1	20.9	50.05	32.83	7.76	8.18	0.6	0.26	10.95	0.79	1.11	3.12	23.26
05-04-2011	Far	D	LC	0.1	20.9	50.05	32.83	7.76	8.18	0.6	0.26	10.43	0.43	0.42	5.4	25.52
05-04-2011	Far	D	LC	4	20.85	50.67	33.29	7.38	8.18	0.6	0.22	0	1.43	1.98	4.23	NA
05-04-2011	Far	D	LC	4	20.85	50.67	33.29	7.38	8.18	0.6	0.22	0	1.35	0.39	4.34	NA
05-04-2011	Far	E	LC	0.1	21.29	48.83	31.93	7.7	8.16	0.5	0.47	14.19	0	0	6.16	41.92
05-04-2011	Far	E	LC	0.1	21.29	48.83	31.93	7.7	8.16	0.5	0.46	14.34	0	0.43	6.92	34.54
05-04-2011	Far	E	LC	3.2	21.24	48.84	31.94	7.39	8.16	0.5	0.47	14.18	0	0	8.08	NA
05-04-2011	Far	E	LC	3.2	21.24	48.84	31.94	7.39	8.16	0.5	0.47	14.23	0	0	7.61	NA
05-04-2011	Near	F	LC	0.1	19.74	49.1	32.15	8.17	8.14	0.4	1.13	30.01	2.61	0.93	7.74	35.06
05-04-2011	Near	F	LC	0.1	19.74	49.1	32.15	8.17	8.14	0.4	1.15	30.71	2.73	0.63	5.93	26.6
05-04-2011	Near	F	LC	1.3	18.94	49.37	32.35	7.91	8.16	0.4	1.03	28.4	2.56	0.69	6.99	NA
05-04-2011	Near	F	LC	1.3	18.94	49.37	32.35	7.91	8.16	0.4	1.08	30.08	2.3	0.3	7.36	NA
05-04-2011	Near	FD	LC	0.1	22.4	42.57	27.34	8.46	8.12	0.6	3.87	89.93	1.04	15.93	6.74	NA
05-04-2011	Near	FD	LC	0.1	22.4	42.57	27.34	8.46	8.12	0.6	3.63	93.83	1.09	11.79	7.28	NA
05-04-2011	Near	FD	LC	0.7	22.1	43.16	27.83	8.61	8.15	0.6	2.37	72.9	1.24	8.33	6.54	NA
05-04-2011	Near	FD	LC	0.7	22.1	43.16	27.83	8.61	8.15	0.6	2.85	77.72	0.62	11.15	7.06	NA
12-07-2011	Far	15	LC	0.1	31.29	49.86	32.45	6.51	8.14	0.2	0.93	44.54	0	0.56	12.82	NA
12-07-2011	Far	15	LC	0.1	31.29	49.86	32.45	6.51	8.14	0.2	1.22	56.29	0.54	0.84	14.94	NA
12-07-2011	Far	15	LC	0.8	31.27	49.84	32.44	6.45	8.13	0.2	1.15	53.28	0.5	0.38	11.33	NA
12-07-2011	Far	15	LC	0.8	31.27	49.84	32.44	6.45	8.13	0.2	1.24	56.7	0.51	0.36	8.37	NA
12-07-2011	Far	8	LC	0.1	30.85	51	33.3	6.21	8.22	0.5	1.01	46.66	0.1	0.53	9.59	NA
12-07-2011	Far	8	LC	0.1	30.85	51	33.3	6.21	8.22	0.5	1.1	51.1	0	0.29	8.99	NA
12-07-2011	Far	8	LC	2.7	30.68	50.94	33.26	6.01	8.21	0.5	0.1	44.55	0.34	0.27	12.3	NA
12-07-2011	Far	8	LC	2.7	30.68	50.94	33.26	6.01	8.21	0.5	1.2	58.53	0.52	0	13.48	NA
12-07-2011	Near	A	LC	0.1	31.76	51.52	33.65	6.46	8.06	0.4	0.42	57.91	0.27	1.09	11.11	65.76
12-07-2011	Near	A	LC	0.1	31.76	51.52	33.65	6.46	8.06	0.4	0.45	58.08	0.3	0.83	8.49	61.16
12-07-2011	Near	A	LC	1.3	31.79	51.5	33.64	6.42	8.06	0.4	0.42	58.33	0.28	0.32	11.89	NA
12-07-2011	Near	A	LC	1.3	31.79	51.5	33.64	6.42	8.06	0.4	0.44	58.63	0.3	0.32	11.5	NA
12-07-2011	Near	B	LC	0.1	31.4	51.34	33.63	7.14	8.22	0.6	0.23	37.72	0	1.04	10.98	34
12-07-2011	Near	B	LC	0.1	31.4	51.34	33.63	7.14	8.22	0.6	0.23	37.46	0	0	9.79	51.5
12-07-2011	Near	B	LC	1.9	31.4	51.29	33.49	7.08	8.22	0.6	0.23	34.07	0	0	7.37	NA
12-07-2011	Near	B	LC	1.9	31.4	51.29	33.49	7.08	8.22	0.6	0.54	38.63	0.64	0.68	0.13	NA
12-07-2011	Far	C	LC	0.1	30.65	50.92	33.33	6.12	8.19	0.7	0.18	26.86	0.46	1.28	6.84	32
12-07-2011	Far	C	LC	0.1	30.65	50.92	33.33	6.12	8.19	0.7	0.17	24.32	0.69	0.48	7.46	22.8
12-07-2011	Far	C	LC	2.9	30.57	51.26	33.51	5.9	8.21	0.7	0.2	29.88	0.35	0	10.88	NA
12-07-2011	Far	C	LC	2.9	30.57	51.26	33.51	5.9	8.21	0.7	0.19	25.18	0.82	0	5.82	NA
12-07-2011	Far	D	LC	0.1	30.23	52.37	34.33	6.39	8.22	0.5	0.22	19.1	0.27	0.94	5.2	20.8
12-07-2011	Far	D	LC	0.1	30.23	52.37	34.33	6.39	8.22	0.5	0.22	18.14	0	0.27	5.05	18.57143
12-07-2011	Far	D	LC	4.4	30.21	53.02	34.83	5.99	8.14	0.5	0.12	0	0	0	5.97	NA
12-07-2011	Far	D	LC	4.4	30.21	53.02	34.83	5.99	8.14	0.5	0.12	0	0.31	0	6.29	NA
12-07-2011	Far	E	LC	0.1	30.65	50.64	33.04	6.29	8.24	0.6	0.6	39.31	0	0.84	9.7	88.4
12-07-2011	Far	E	LC	0.1	30.65	50.64	33.04	6.29	8.24	0.6	0.77	50.7	0.35	0.52	11.61	87.48
12-07-2011	Far	E	LC	3.4	30.47	50.56	32.99	5.95	8.23	0.6	0.71	44.51	0	0	7.51	NA
12-07-2011	Far	E	LC	3.4	30.47	50.56	32.99	5.95	8.23	0.6	0.76	46.59	0	0	5.64	NA
12-07-2011	Near	F	LC	0.1	31	51.82	33.87	6.37	8.23	0.4	1.16	48.27	0	1.06	11.19	45.76
12-07-2011	Near	F	LC	0.1	31	51.82	33.87	6.37	8.23	0.4	1.29	55.39	0.63	1.33	13.28	44.56
12-07-2011	Near	F	LC	1.5	30.79	51.82	33.89	6.26	8.23	0.4	1.19	51.29	0.3	0.41	11.68	NA
12-07-2011	Near	F	LC	1.5	30.79	51.82	33.89	6.26	8.23	0.4	0.128	55.75	0.38	0.41	12.91	NA
12-07-2011	Near	FD	LC	0.1	31.83	51.34	33.51	6.93	8.11	0.3	0.41	59.77	0.26	1.37	7.07	NA
12-07-2011	Near	FD	LC	0.1	31.83	51.34	33.51	6.93	8.11	0.3	0.41	58.07	0	1.16	7.95	NA
12-07-2011	Near	FD	LC	0.8	31.84	51.32	33.5	6.93	8.11	0.3	0.43	60.88	0.3	0.33	13.05	NA
12-07-2011	Near	FD	LC	0.8	31.84	51.32	33.5	6.93	8.11	0.3	0.43	60.04	0	0	8.19	NA
04-10-2011	Far	15	LC	0.1	24.32	57.6	34.68	8.46	8.21	0.5	2.29	84.61	0	1.03	14.52	NA
04-10-2011	Far	15	LC	0.1	24.32	57.6	34.68	8.46	8.21	0.5	2.42	74.45	0	0.53	11.08	NA
04-10-2011	Far	15	LC	0.6	23.34	57.8	38.72	7.51	8.19	0.5	2.4	73.39	0	0.34	9.23	NA
04-10-2011	Far	15	LC	0.6	23.34	57.8	38.72	7.51	8.19	0.5	2.72	74.55	0	0.3	11.29	NA
04-10-2011	Far	8	LC	0.1	24.97	58.61	39.16	7.63	8.19	0.7	1.67	58.81	0	1.05	12.35	NA
04-10-2011	Far	8	LC	0.1	24.97	58.61	39.16	7.63	8.19	0.7	1.7	57.77	0	0.28	9.18	NA
04-10-2011	Far	8	LC	2.5	24.74	58.61	39.16	7.4	8.19	0.7	1.71	58.26	0	2.71	14.28	NA
04-10-2011	Far	8	LC	2.5	24.74	58.61	39.16	7.4	8.19	0.7	1.71	59.48	0	0.33	13.52	NA
04-10-2011	Near	A	LC	0.1	24.86	59.99	40.21	7.53	8.04	0.4	0.46	95.44	0	1.44	13.4	87.6
04-10-2011	Near	A	LC	0.1	24.86	59.99	40.21	7.53	8.04	0.4	0.47	96.58	0	0.52	13.7	53.66667
04-10-2011	Near	A	LC	1.1	24.88	60	40.21	7.57	8.04	0.4	0.35	106.11	0	0.33	8.93	NA
04-10-2011	Near	A	LC	1.1	24.88	60	40.21	7.57	8.04	0.4	0.42	97.28	0	0.28	9.45	NA
04-10-2011	Near	B	LC	0.1	24.86	60.09	40.28	7.23	8.05	0.7	0.28	63.78	0	1.12	6.98	120
04-10-2011	Near	B	LC	0.1	24.86	60.09	40.28	7.23	8.05	0.7	0.28	61.15	0	0	10.224	152.96
04-10-2011	Near	B	LC	1.7	24.6	60.19	40.35	6.98	8.02	0.7	0.29	62.48	0	0.34	6.06	NA
04-10-2011	Near	B	LC	1.7	24.6	60.19	40.35	6.98	8.02	0.7	0.28	64.4	0	0.29	9.27	NA
04-10-2011	Far	C	LC	0.1	24.56	58.34	39.02	6.37	8.05	0.5	0.46	46.46	0	0.86	9.83	163.05
04-10-2011	Far	C	LC	0.1	24.56	58.34	39.02	6.37	8.05	0.5	0.47	46.14	0	0	6	64.75
04-10-2011	Far	C	LC	2.7	24.55	58.48	39.07	6.26	8.06	0.5	0.45	45.35	0	0.25	11.79	NA
04-10-2011	Far	C	LC	2.7	24.55	58.48	39.07	6.26	8.06	0.5	0.4	40.79	0	0	6.41	NA
04-10-2011	Far	D	LC	0.1	25.41	57.4	38.23	6.98	8.15	1.4	0.37	22.12	0	0.84	4.43	25.7
04-10-2011	Far	D	LC	0.1	25.41	57.4	38.23	6.98	8.15	1.4	0.39	21.33	0	4.03	2.05	13.9
04-10-2011	Far	D	LC	3.7	25.3	57.45	38.25	6.66	8.16	1.4	0.4	21.85	0	0.62	4.76	NA
04-10-2011	Far	D	LC	3.7	25.3	57.45	38.25	6.66	8.16	1.4	0.39	21.06	0	0.43	3.44	NA
04-10-2011	Far	E	LC	0.1	25.05	57.95	38.65	6.74	8.16	0.6	0.66	39.19	0	1.19	0.23	37.9
04-10-2011	Far	E	LC	0.1	25.05	57.95	38.65	6.74	8.16	0.6	0.73	37.02	0	41.09	5.62	33.23333
04-10-2011	Far	E	LC	3.2	24.82	57.88	38.61	6.49	8.16	0.6	0.64	37.58	0	0.42	7.47	NA
04-10-2011	Far	E	LC	3.2	24.82	57.88	38.61	6.49	8.16	0.6	0.69	38.57	0	0.43	8.66	NA
04-10-2011	Near	F	LC	0.1	23.8	57.9	38.66	7.07	8.16	0.6	2.1	67.88	0	1.36	8.71	36.3
04-10-2011	Near	F	LC	0.1	23.8	57.9	38.66	7.07	8.16	0.6	1.79	60.95	0	0.43	6.93	30.96667
04-10-2011	Near	F	LC	1.3	23.55	58.16	38.85	6.9	8.15	0.6	2.06	65.86	0	0.37	5.94	NA
04-10-2011	Near	F	LC	1.3	23.55	58.16	38.85	6.9	8.15	0.6	1.99	66.03	0	0.29	5.54	NA
04-10-2011	Near	FD	LC	0.1	26.5	60.9	40.87	7.57	8.03	0.4	0.32	91.98	0	0.29	8.27	NA
04-10-2011	Near	FD	LC	0.1	26.5	60.9	40.87	7.57	8.03	0.4	0.27	83.32	0	0.3	11.68	NA
04-10-2011	Near	FD	LC	1	26.5	60.94	40.88	7.6	8.04	0.4	0.23	58.91	0	0.34	6.74	NA
04-10-2011	Near	FD	LC	1	26.5	60.94	40.88	7.6	8.04	0.4	0.29	85.31	0	0.32	8.7	NA
05-01-2012	Far	15	LC	0.1	15.19		23	15.31	8.57	0.6	3.61	18.55	1.47	51.41	5.29	NA
05-01-2012	Far	15	LC	0.1	15.19		23	15.31	8.57	0.6	3.74	19.28	1.65	51.28	4.82	NA
05-01-2012	Far	15	LC	0.5	14.59		30	15.62	8.58	0.6	0.68	0	0.36	5.85	10.49	NA
05-01-2012	Far	15	LC	0.5	14.59		30	15.62	8.58	0.6	0.69	0	0.82	6.28	9.98	NA
05-01-2012	Far	8	LC	0.1	14.48		30	11.87	8.43	1.2	0.18	0	0.27	0.28	7.36	NA
05-01-2012	Far	8	LC	0.1	14.48		30	11.87	8.43	1.2	0.14	0	1.04	0	7.02	NA
05-01-2012	Far	8	LC	1.8	12.92		32	10.1	8.3	1.2	0.29	0	0.38	0	6.49	NA
05-01-2012	Far	8	LC	1.8	12.92		32	10.1	8.3	1.2	0.47	0	0.36	0	2.63	NA
05-01-2012	Near	A	LC	0.1	15.47		36.44	8.83	8.13	1.2	0.81	22.48	1.39	1.32	0.24	15.825
05-01-2012	Near	A	LC	0.1	15.47		36.44	8.83	8.13	1.2	0.83	22.07	1.39	1.4	0.27	18.75
05-01-2012	Near	A	LC	0.9	15.47		36	8.87	8.13	1.2	0.81	22.8	1.19	1.23	0.23	NA
05-01-2012	Near	A	LC	0.9	15.47		36	8.87	8.13	1.2	0.82	22.98	0.86	1.09	0.17	NA
05-01-2012	Near	B	LC	0.1	14.8		35	9.17	8.2	1.7	0.37	0	0.39	1	0.55	15
05-01-2012	Near	B	LC	0.1	14.8		35	9.17	8.2	1.7	0.32	0	0.46	0	0.63	14.7
05-01-2012	Near	B	LC	1.4	14.43		35	9.28	8.17	1.7	0.56	11.91	0	0.43	0.36	NA
05-01-2012	Near	B	LC	1.4	14.43		35	9.28	8.17	1.7	0.57	12.07	0.48	0.56	0.38	NA
05-01-2012	Far	C	LC	0.1	13.42		34	8.88	8.07	1.2	0.23	0	0.26	0.26	1.17	17.8
05-01-2012	Far	C	LC	0.1	13.42		34	8.88	8.07	1.2	0.21	0	0	0	1.15	16.74286
05-01-2012	Far	C	LC	2.2	13.38		34	8.87	8.09	1.2	0.34	0	0.51	0	1.13	NA
05-01-2012	Far	C	LC	2.2	13.38		34	8.87	8.09	1.2	0.49	0	0.25	0	1.14	NA
05-01-2012	Far	D	LC	0.1	13.2		31	9.28	8.18	1.1	0.16	0	0.32	0	1.07	16.96667
05-01-2012	Far	D	LC	0.1	13.2		31	9.28	8.18	1.1	0.16	0	0	0	0.98	17.86667
05-01-2012	Far	D	LC	3.5	13.15		32	9.42	8.2	1.1	0.42	0	0	0	1.22	NA
05-01-2012	Far	D	LC	3.5	13.15		32	9.42	8.2	1.1	0.54	0	0.25	0	1.85	NA
05-01-2012	Far	E	LC	0.1	14.36		30	10.68	8.34	1	0.25	0	0.25	0.75	2.4	16.16667
05-01-2012	Far	E	LC	0.1	14.36		30	10.68	8.34	1	0.25	0	0.51	0	4.99	16
05-01-2012	Far	E	LC	2.9	13.3		32	9.82	8.27	1	0.43	0	0	0	3.22	NA
05-01-2012	Far	E	LC	2.9	13.3		32	9.82	8.27	1	0.48	0	0.52	0	2.9	NA
05-01-2012	Near	F	LC	0.1	15.65		27	12.22	8.55	0.7	0.41	0	0.46	2.52	8.04	16.4
05-01-2012	Near	F	LC	0.1	15.65		27	12.22	8.55	0.7	0.43	0	0.53	3.42	9.63	18.23333
05-01-2012	Near	F	LC	1	13.84		30	13.76	8.52	0.7	0.33	0	0.41	0	8.89	NA
05-01-2012	Near	F	LC	1	13.84		30	13.76	8.52	0.7	0.32	0	0.66	0	9.55	NA
05-01-2012	Near	FD	LC	0.1	16.93		36.18	9.51	8.09	0.6	3.56	73.9	2.3	13.3	0.38	NA
05-01-2012	Near	FD	LC	0.1	16.93		36.18	9.51	8.09	0.6	2.58	48.08	1.85	6.09	0.35	NA
05-01-2012	Near	FD	LC	0.6	16.31		36	9.92	8.14	0.6	2.28	44.74	2.13	3.11	0.32	NA
05-01-2012	Near	FD	LC	0.6	16.31		36	9.92	8.14	0.6	0.95	25.51	1.32	1.34	0.27	NA
11-04-2012	Far	15	LC	0.1	27.01	31.5	19.62	9.88	8.6	0.2	0	27.69	0.97	0.39	23.37	NA
11-04-2012	Far	15	LC	0.1	27.01	31.5	19.62	9.88	8.6	0.2	0	20.42	2.64	0	16.75	NA
11-04-2012	Far	15	LC	1.1	27	31.6	19.64	9.75	8.59	0.2	0	21.45	2.35	0	10.89	NA
11-04-2012	Far	15	LC	1.1	27	31.6	19.64	9.75	8.59	0.2	0	19.88	2.26	0	14.37	NA
11-04-2012	Far	8	LC	0.1	26.07	39.3	25.09	8.66	8.14	1	0	10.6	1.57	0	4.13	NA
11-04-2012	Far	8	LC	0.1	26.07	39.3	25.09	8.66	8.14	1	0	0	1.46	0	3.74	NA
11-04-2012	Far	8	LC	2.1	25.93	39.3	25.05	8.46	8.15	1	0	0	1.17	0	3.18	NA
11-04-2012	Far	8	LC	2.1	25.93	39.3	25.05	8.46	8.15	1	0	0	1.24	0	4.72	NA
11-04-2012	Near	A	LC	0.1	24.94	34.7	21.77	7.71	8.04	0.4	0.15	49.49	3.79	0.95	3.56	44.8
11-04-2012	Near	A	LC	0.1	24.94	34.7	21.77	7.71	8.04	0.4	0.14	47.58	3.47	0.4	4.7	42.33333
11-04-2012	Near	A	LC	1.3	24.93	34.7	21.81	7.67	8.04	0.4	0.18	44.14	4.15	0	5.7	NA
11-04-2012	Near	A	LC	1.3	24.93	34.7	21.81	7.67	8.04	0.4	0.17	43.48	3.76	0.44	5.79	NA
11-04-2012	Near	B	LC	0.1	24.97	38.1	24.22	7.41	8.01	0.3	0.24	31.01	4.47	0.65	3.71	60.33333
11-04-2012	Near	B	LC	0.1	24.97	38.1	24.22	7.41	8.01	0.3	0.22	28.3	4.5	0.52	4.93	63.2
11-04-2012	Near	B	LC	1.6	24.95	38.1	24.22	7.38	8.01	0.3	0.22	29.06	4.64	0.38	4.49	NA
11-04-2012	Near	B	LC	1.6	24.95	38.1	24.22	7.38	8.01	0.3	0.24	31.08	4.42	0.47	4.55	NA
11-04-2012	Far	C	LC	0.1	25.69	38.21	24.24	6.93	8.02	0.8	0	14.6	1.25	0.86	4.66	19.13333
11-04-2012	Far	C	LC	0.1	25.69	38.21	24.24	6.93	8.02	0.8	0	13.47	1.62	0	4.51	21.83333
11-04-2012	Far	C	LC	2.8	25.62	38.4	24.38	6.84	8.01	0.8	0	15.37	1.37	0	3.75	NA
11-04-2012	Far	C	LC	2.8	25.62	38.4	24.38	6.84	8.01	0.8	0	14.9	1.61	0	3.58	NA
11-04-2012	Far	D	LC	0.1	25.5	39.28	25	6.59	7.98	0.8	0.11	19.51	1.08	0	4.1	25.03333
11-04-2012	Far	D	LC	0.1	25.5	39.28	25	6.59	7.98	0.8	0.11	15.53	1.31	0	4.13	26.4
11-04-2012	Far	D	LC	4.1	24.9	40.17	25.65	6.47	7.94	0.8	0.16	15.06	2.65	0	4.96	NA
11-04-2012	Far	D	LC	4.1	24.9	40.17	25.65	6.47	7.94	0.8	0.16	17.69	2.15	0	4.58	NA
11-04-2012	Far	E	LC	0.1	25.94	39.5	25.18	8.4	8.14	0.7	0	12.03	2.07	0	7.89	20.03333
11-04-2012	Far	E	LC	0.1	25.94	39.5	25.18	8.4	8.14	0.7	0	16.29	2.19	0	7.59	22.52
11-04-2012	Far	E	LC	3	25.72	39.5	25.19	7.75	8.15	0.7	0	13.56	1.28	0	7.26	NA
11-04-2012	Far	E	LC	3	25.72	39.5	25.19	7.75	8.15	0.7	0	12.1	1.75	0	4.94	NA
11-04-2012	Near	F	LC	0.1	26.47	36	22.77	9.35	8.32	0.5	0	0	2.57	0	9.99	28.4
11-04-2012	Near	F	LC	0.1	26.47	36	22.77	9.35	8.32	0.5	0	0	2.24	0	8.98	30.03333
11-04-2012	Near	F	LC	1.8	26.44	36.2	22.8	9.15	8.32	0.5	0	0	2.03	0	7.09	NA
11-04-2012	Near	F	LC	1.8	26.44	36.2	22.8	9.15	8.32	0.5	0	0	1.63	0	10.61	NA
11-04-2012	Near	FD	LC	0.1	26.22	35.2	22.14	6.78	7.85	0.4	5.56	131.88	5.48	57.6	4.22	NA
11-04-2012	Near	FD	LC	0.1	26.22	35.2	22.14	6.78	7.85	0.4	5.9	135.44	5.42	63.7	3.9	NA
11-04-2012	Near	FD	LC	0.9	25.58	35.2	22.15	7.21	7.87	0.4	2.44	83.3	8.84	28.6	5.46	NA
11-04-2012	Near	FD	LC	0.9	25.58	35.2	22.15	7.21	7.87	0.4	2.9	90.93	5.04	31.3	5.3	NA
11-07-2012	Far	15	LC	0.1	26.83	43.91	28.28	6.72	8.19	0.3	1.67	57.12	6.7	2.34	9.33	NA
11-07-2012	Far	15	LC	0.1	26.83	43.91	28.28	6.72	8.19	0.3	1.72	55.56	6.01	3.46	11.11	NA
11-07-2012	Far	15	LC	0.7	26.57	43.92	28.29	6.6	8.19	0.3	1.71	55.89	5.84	1.94	8.82	NA
11-07-2012	Far	15	LC	0.7	26.57	43.92	28.29	6.6	8.19	0.3	1.69	54.65	5.95	2.66	10.78	NA
11-07-2012	Far	8	LC	0.1	27.32	44.26	28.51	6.23	8.24	0.4	1.01	51.41	1.57	0.44	15.25	NA
11-07-2012	Far	8	LC	0.1	27.32	44.26	28.51	6.23	8.24	0.4	1.18	54.83	2	0.84	7.53	NA
11-07-2012	Far	8	LC	2	27.35	44.36	28.59	6.09	8.24	0.4	1.18	57.63	1.53	0.55	9.15	NA
11-07-2012	Far	8	LC	2	27.35	44.36	28.59	6.09	8.24	0.4	1.19	57.22	1.52	0.97	3.4	NA
11-07-2012	Near	A	LC	0.1	26.99	30.45	18.95	7.12	8.14	0.4	1.21	151.8	6.55	1.36	26.11	44.05
11-07-2012	Near	A	LC	0.1	26.99	30.45	18.95	7.12	8.14	0.4	1.28	169.24	7.15	1.35	22.47	39.25
11-07-2012	Near	A	LC	1	26.44	36.65	23.04	6.82	8.1	0.4	0.43	104.46	4.89	2.1	7.73	NA
11-07-2012	Near	A	LC	1	26.44	36.65	23.04	6.82	8.1	0.4	0.45	94.18	5.32	1.95	10.69	NA
11-07-2012	Near	B	LC	0.1	26.93	36.08	22.73	6.46	8.12	0.3	0.28	111.49	6.23	1.93	6.82	67.8
11-07-2012	Near	B	LC	0.1	26.93	36.08	22.73	6.46	8.12	0.3	0.28	105.14	5.93	2.37	9.58	62.52
11-07-2012	Near	B	LC	1.6	26.94	36.17	22.79	6.31	8.11	0.3	0.28	101.74	6.3	1.67	3.68	NA
11-07-2012	Near	B	LC	1.6	26.94	36.17	22.79	6.31	8.11	0.3	0.28	101.8	6.3	1.64	9.28	NA
11-07-2012	Far	C	LC	0.1	27.83	44.42	28.61	6.13	8.11	0.4	0.37	51.02	5.55	1.01	3.87	39.65
11-07-2012	Far	C	LC	0.1	27.83	44.42	28.61	6.13	8.11	0.4	0.4	53.92	5.8	1.14	4.95	42.3
11-07-2012	Far	C	LC	2.3	27.95	44.92	28.95	5.96	8.13	0.4	0.34	50.53	5.56	1.15	2.29	42.3
11-07-2012	Far	C	LC	2.3	27.95	44.92	28.95	5.96	8.13	0.4	0.37	52.15	5.25	0.94	4.49	42.3
11-07-2012	Far	D	LC	0.1	27.9	46.93	30.92	6.06	8.21	0.5	0.58	55.53	4.62	1.47	1.2	35.4
11-07-2012	Far	D	LC	0.1	27.9	46.93	30.92	6.06	8.21	0.5	0.6	56.92	4.18	0.82	4.82	36.1
11-07-2012	Far	D	LC	3.6	27.43	46.94	30.43	5.92	8.22	0.5	0.6	55.97	4.16	0.61	6.87	NA
11-07-2012	Far	D	LC	3.6	27.43	46.94	30.43	5.92	8.22	0.5	0.63	58.37	4.46	1.58	5.96	NA
11-07-2012	Far	E	LC	0.1	27.72	45.85	29.64	6.27	8.25	0.4	0.62	50.95	2.95	0.6	5.16	58.65
11-07-2012	Far	E	LC	0.1	27.72	45.85	29.64	6.27	8.25	0.4	0.62	51.25	2.96	0.66	5.09	59.05
11-07-2012	Far	E	LC	3	27.75	46.17	29.88	6.14	8.25	0.4	0.62	52.96	2.91	0.47	4.62	NA
11-07-2012	Far	E	LC	3	27.75	46.17	29.88	6.14	8.25	0.4	0.62	53.86	2.96	1.24	4.84	NA
11-07-2012	Near	F	LC	0.1	26.81	44.7	28.84	6.46	8.26	0.4	1.22	48.6	1.4	0.71	9.64	68
11-07-2012	Near	F	LC	0.1	26.81	44.7	28.84	6.46	8.26	0.4	1.24	50.19	1.42	0.82	7.67	66.1
11-07-2012	Near	F	LC	1.3	26.84	44.75	28.88	6.37	8.26	0.4	1.26	47.42	1.82	0.35	8.41	NA
11-07-2012	Near	F	LC	1.3	26.84	44.75	28.88	6.37	8.26	0.4	1.26	51.12	2.01	0.89	7.84	NA
11-07-2012	Near	FD	LC	0.1	27.62	26.95	16.33	5.76	8.03	0.3	1.73	151.44	10.36	1.06	23.36	NA
11-07-2012	Near	FD	LC	0.1	27.62	26.95	16.33	5.76	8.03	0.3	2.03	179.22	11.05	1.31	20.43	NA
11-07-2012	Near	FD	LC	0.8	27.09	31.22	19.9	6.13	8.05	0.3	1.97	156.65	10.34	5.14	16.15	NA
11-07-2012	Near	FD	LC	0.8	27.09	31.22	19.9	6.13	8.05	0.3	2.02	172.68	9.98	5.12	17.01	NA
03-10-2012	Far	15	LC	0.1	26.6	35.55	22.5	8.9	8.22	0.5	3.4	101.26	1.99	14.08	9.49	NA
03-10-2012	Far	15	LC	0.1	26.6	35.55	22.5	8.9	8.22	0.5	3.13	94.23	0.65	8.03	15.29	NA
03-10-2012	Far	15	LC	0.6	26.6	35.55	22.5	8.9	8.22	0.5	2.88	95.27	0.7	3.19	15.29	NA
03-10-2012	Far	15	LC	0.6	26.6	35.55	22.5	8.9	8.22	0.5	2.89	94.06	0.69	3.51	15.29	NA
03-10-2012	Far	8	LC	0.1	24.66	43.68	28.16	8.18	8.24	0.9	1.96	89.9	0	1.09	12.26	NA
03-10-2012	Far	8	LC	0.1	24.66	43.68	28.16	8.18	8.24	0.9	1.83	86.98	0.94	0.62	8.99	NA
03-10-2012	Far	8	LC	2.4	23.78	45.91	29.78	7.68	8.27	0.9	1.63	82.6	1.21	0.41	12.34	NA
03-10-2012	Far	8	LC	2.4	23.78	45.91	29.78	7.68	8.27	0.9	1.67	81.91	0.8	0.76	11.77	NA
03-10-2012	Near	A	LC	0.1	26.34	32.75	20.42	9.22	8.31	0.6	0.99	146.2	1.64	2.32	10.76	25.73333
03-10-2012	Near	A	LC	0.1	26.34	32.75	20.42	9.22	8.31	0.6	1	126.71	1.26	0.81	10.9	28.96667
03-10-2012	Near	A	LC	1.1	26.13	34.19	21.29	8.85	8.28	0.6	1.02	138.18	1.04	0	8.36	NA
03-10-2012	Near	A	LC	1.1	26.13	34.19	21.29	8.85	8.28	0.6	1	134.08	1.65	0.27	7.9	NA
03-10-2012	Near	B	LC	0.1	25.73	37.01	23.41	8.81	8.28	0.9	1.08	117.15	0.66	0	13.36	24.73333
03-10-2012	Near	B	LC	0.1	25.73	37.01	23.41	8.81	8.28	0.9	0.91	87.62	0.38	0.92	9.18	22.13333
03-10-2012	Near	B	LC	1.6	25.46	43.06	27.65	7	8.1	0.9	0.89	102.81	0.92	0.85	11.87	NA
03-10-2012	Near	B	LC	1.6	25.46	43.06	27.65	7	8.1	0.9	0.91	94.38	0.66	0	14.53	NA
03-10-2012	Far	C	LC	0.1	23.73	40.94	26.21	7.58	8.17	0.9	1.08	91.08	1.94	4.59	5.63	30.93333
03-10-2012	Far	C	LC	0.1	23.73	40.94	26.21	7.58	8.17	0.9	1.1	82.4	1.13	3.94	7.19	21
03-10-2012	Far	C	LC	2.7	24.99	47.7	31.05	6.74	8.18	0.9	0.05	69.22	0	0.31	6.94	NA
03-10-2012	Far	C	LC	2.7	24.99	47.7	31.05	6.74	8.18	0.9	0.05	67.39	1.49	0	9.7	NA
03-10-2012	Far	D	LC	0.1	25.16	47.07	30.58	7.5	8.23	1	1	74.24	0.99	4.09	7.54	15.775
03-10-2012	Far	D	LC	0.1	25.16	47.07	30.58	7.5	8.23	1	1.03	71.93	0.86	0.151	7.01	13.475
03-10-2012	Far	D	LC	3.9	26.16	53.47	35.27	5.89	8.15	1	0.61	30.78	0.94	0.4	5.46	NA
03-10-2012	Far	D	LC	3.9	26.16	53.47	35.27	5.89	8.15	1	0.59	31.5	1.86	0.51	6.83	NA
03-10-2012	Far	E	LC	0.1	24.71	43.62	28.11	8.33	8.29	0.9	1.09	78.62	0.62	1.83	17.88	21.4
03-10-2012	Far	E	LC	0.1	24.71	43.62	28.11	8.33	8.29	0.9	1.11	80.88	0.72	0.27	13.62	15.43333
03-10-2012	Far	E	LC	3.2	24.23	45.13	29.18	5.75	8.16	0.9	1.21	75.86	0.25	0.4	18.53	NA
03-10-2012	Far	E	LC	3.2	24.23	45.13	29.18	5.75	8.16	0.9	1.19	81.41	1.73	0	16.911	NA
03-10-2012	Near	F	LC	0.1	25.62	37.88	24.03	8.99	8.25	0.5	2.03	94.23	1.65	2.94	15.25	57.91429
03-10-2012	Near	F	LC	0.1	25.62	37.88	24.03	8.99	8.25	0.5	1.96	90.27	0.6	0.59	17.54	58.57143
03-10-2012	Near	F	LC	1.2	23.55	41.07	26.31	7.86	8.2	0.5	2.28	89.74	0.63	0.65	17.8	NA
03-10-2012	Near	F	LC	1.2	23.55	41.07	26.31	7.86	8.2	0.5	2.27	91.78	0.73	0.8	15.22	NA
03-10-2012	Near	FD	LC	0.1	26.63	30.41	18.83	9.34	8.33	0.5	1.11	138.81	1.63	1.89	12.74	NA
03-10-2012	Near	FD	LC	0.1	26.63	30.41	18.83	9.34	8.33	0.5	1.12	147.73	1.58	1.24	11.11	NA
03-10-2012	Near	FD	LC	0.7	26.62	30.41	18.83	9.39	8.33	0.5	1.15	162.18	1.58	0	12.72	NA
03-10-2012	Near	FD	LC	0.7	26.62	30.41	18.83	9.39	8.33	0.5	1.15	163.22	1.69	0	21.77	NA
10-01-2013	Far	15	LC	0.1	15.36	38.07	24.22	10.26	8.11	1	4.26	34.29	10.01	34.28	1.86	28.4
10-01-2013	Far	15	LC	0.1	15.36	38.07	24.22	10.26	8.11	1	4.28	34.43	10.55	35.03	1.77	26.74286
10-01-2013	Far	15	LC	0.6	14.98	38.83	24.76	11.11	8.13	1	3.86	32.82	8.38	31.6	3.07	NA
10-01-2013	Far	15	LC	0.6	14.98	38.83	24.76	11.11	8.13	1	3.9	31.85	7.97	32.18	2.41	NA
10-01-2013	Far	8	LC	0.1	13.23	46.29	30.03	9.04	8.13	0.4	1.41	38.48	2.8	3.91	3.54	93.68571
10-01-2013	Far	8	LC	0.1	13.23	46.29	30.03	9.04	8.13	0.4	1.41	36.57	4.29	4.4	4	96.2
10-01-2013	Far	8	LC	2.3	12.79	46.33	30.04	8.89	8.13	0.4	1.42	37.04	2.31	3.69	4.86	NA
10-01-2013	Far	8	LC	2.3	12.79	46.33	30.04	8.89	8.13	0.4	1.38	38.37	6.78	3.5	5.52	NA
10-01-2013	Near	A	LC	0.1	15.52	40.59	26	9.54	8.2	1.4	0.66	56.2	2.73	1.07	3.23	18.31429
10-01-2013	Near	A	LC	0.1	15.52	40.59	26	9.54	8.2	1.4	0.69	56.15	0.65	1.21	4.73	25.74286
10-01-2013	Near	A	LC	1	15.52	40.59	26	9.54	8.2	1.4	0.66	56.2	2.73	1.07	4.73	11.57143
10-01-2013	Near	A	LC	1	15.52	40.59	26	9.54	8.2	1.4	0.69	56.15	0.65	1.21	4.73	11.17143
10-01-2013	Near	B	LC	0.1	14.16	42.73	27.5	9.64	8.23	1.9	0.65	37.9	3.93	1.21	2.73	16.51429
10-01-2013	Near	B	LC	0.1	14.16	42.73	27.5	9.64	8.23	1.9	0.63	40.15	1.92	1.36	2.04	13.85714
10-01-2013	Near	B	LC	1.6	14.16	42.73	27.5	9.64	8.23	1.9	0.62	40.91	1.92	1.36	2.04	12.45714
10-01-2013	Near	B	LC	1.6	14.16	42.73	27.5	9.64	8.23	1.9	0.65	37.9	3.93	1.21	2.04	9.657143
10-01-2013	Far	C	LC	0.1	12.19	47.48	30.82	8.96	8.1	0.7	0.45	25.34	0.33	1	5.03	31.57143
10-01-2013	Far	C	LC	0.1	12.19	47.48	30.82	8.96	8.1	0.7	0.44	24.69	1.62	0.95	5.58	30.74286
10-01-2013	Far	C	LC	2.7	12.14	47.4	30.77	8.92	8.08	0.7	0.43	24.95	1.64	0.96	5.58	22.2
10-01-2013	Far	C	LC	2.7	12.14	47.4	30.77	8.92	8.08	0.7	0.45	25.34	0.33	1	5.58	29.48571
10-01-2013	Far	D	LC	0.1	12.12	47.94	31.17	8.96	8.09	0.7	0.78	30.77	2.92	1.74	2.45	21.4
10-01-2013	Far	D	LC	0.1	12.12	47.94	31.17	8.96	8.09	0.7	0.77	30.97	3.79	1.88	2.77	43.94286
10-01-2013	Far	D	LC	3.9	11.83	48.04	31.23	8.9	8.09	0.7	0.78	30.77	2.92	1.74	2.77	28.85714
10-01-2013	Far	D	LC	3.9	11.83	48.04	31.23	8.9	8.09	0.7	0.77	30.97	3.79	1.88	2.77	29.02857
10-01-2013	Far	E	LC	0.1	12.64	47.03	30.54	8.98	8.1	0.5	0.98	33.28	1.56	2.73	2.96	23.77143
10-01-2013	Far	E	LC	0.1	12.64	47.03	30.54	8.98	8.1	0.5	0.99	35.82	1.99	3.03	3.86	24.14286
10-01-2013	Far	E	LC	3.2	12.13	47.13	30.59	8.91	8.1	0.5	0.95	35.1	2.42	2.49	6.36	48.13333
10-01-2013	Far	E	LC	3.2	12.13	47.13	30.59	8.91	8.1	0.5	0.96	34.84	3.07	2.43	2.34	45.23333
10-01-2013	Near	F	LC	0.1	16.04	39.63	25.32	8.52	8.19	1	1.82	16.63	10.06	9.9	1.85	12.82857
10-01-2013	Near	F	LC	0.1	16.04	39.63	25.32	8.52	8.19	1	1.75	16.44	7.38	11.43	1.64	11.25714
10-01-2013	Near	F	LC	1.2	14.14	44.81	28.99	9.39	8.21	1	1.72	16.29	7.38	11.43	5.5	20.97143
10-01-2013	Near	F	LC	1.2	14.14	44.81	28.99	9.39	8.21	1	1.82	16.63	10.06	9.9	4.87	20.02857
10-01-2013	Near	FD	LC	0.1	16.2	38.94	24.91	10.27	8.21	1.1	0.81	57.1	3.04	1.86	3.9	12.85714
10-01-2013	Near	FD	LC	0.1	16.2	38.94	24.91	10.27	8.21	1.1	0.75	68.97	2.33	2.78	0.83	12.85714
10-01-2013	Near	FD	LC	0.7	15.26	41.18	26.42	11.56	8.26	1.1	0.88	52.19	1.34	2.66	3.83	12.85714
10-01-2013	Near	FD	LC	0.7	15.26	41.18	26.42	11.56	8.26	1.1	0.88	64.44	1.29	1.18	4.79	12.85714
16-04-2013	Far	15	LC	0.1	24.28	46.16	29.95	7.03	8.2	0.4	0.47	15.25	1.13	1.12	14.56	82.61
16-04-2013	Far	15	LC	0.1	24.28	46.16	29.95	7.03	8.2	0.4	0.49	16.53	0.43	0.47	14.4	70.23333
16-04-2013	Far	15	LC	0.9	24.28	46.16	29.95	7.03	8.2	0.4	0.49	15.87	5.28	0.26	15.98	55.66667
16-04-2013	Far	15	LC	0.9	24.28	46.16	29.95	7.01	8.2	0.4	0.49	15.98	1.16	0.41	16.23	72.73333
16-04-2013	Far	8	LC	0.1	22.49	47.42	30.9	7.09	8.12	0.6	0.28	12.14	1.1	0.34	5.62	27
16-04-2013	Far	8	LC	0.1	22.49	47.42	30.9	7.09	8.12	0.6	0.28	11.71	2.28	0.5	6.11	34.16667
16-04-2013	Far	8	LC	2.6	22.36	47.47	30.9	7.25	8.11	0.6	0.3	11.65	0.72	0.47	5.3	27.4
16-04-2013	Far	8	LC	2.6	22.36	47.47	30.9	7.25	8.11	0.6	0.29	11.72	1.65	0	6.29	23.23333
16-04-2013	Near	A	LC	0.1	23.94	45.96	29.82	7.65	8.11	0.5	0.13	32.2	0.59	0.6	7.49	36.92
16-04-2013	Near	A	LC	0.1	23.94	45.96	29.82	7.65	8.11	0.5	0.14	32.32	0.63	0.75	6.59	38.2
16-04-2013	Near	A	LC	1.5	23.94	45.96	29.82	7.65	8.11	0.5	0.14	32.72	0.64		6.94	45.68
16-04-2013	Near	A	LC	1.5	23.94	45.98	29.83	7.65	8.11	0.5	0.14	32.22	1.18	0.26	5.99	49.04
16-04-2013	Near	B	LC	0.1	23.75	46.41	30.14	7.23	8.11	0.4	0.15	25.98	2.23	1.07	7.33	73.08
16-04-2013	Near	B	LC	0.1	23.75	46.41	30.14	7.23	8.11	0.4	0.14	25.35	2.58	1.26	6.25	67.92
16-04-2013	Near	B	LC	2.1	23.76	46.39	30.14	7.24	8.1	0.4	0.13	25.39	2.95	0.34	6.56	60.7
16-04-2013	Near	B	LC	2.1	23.76	46.39	30.14	7.24	8.1	0.4	0.14	25.15	2.97	0.31	5.43	50.05
16-04-2013	Far	C	LC	0.1	22.34	47.34	30.84	7.23	8.09	0.6	0.12	14.05	1.94	1.78	6.82	29.92
16-04-2013	Far	C	LC	0.1	22.34	47.34	30.84	7.23	8.09	0.6	0.11	14.24	6.66	0.44	7.62	33.76
16-04-2013	Far	C	LC	2.9	22.42	47.68	31.09	6.96	8.05	0.6	0.13	16.61	3.03	0.25	8.84	48.3
16-04-2013	Far	C	LC	2.9	22.42	47.68	31.09	6.96	8.05	0.6	0.14	16.35	2.29	0.34	8.84	68.55
16-04-2013	Far	D	LC	0.1	22.17	47.09	30.72	7.42	8.11	0.9	0.17		0.98	0.32	1.88	48.23333
16-04-2013	Far	D	LC	0.1	22.17	47.09	30.72	7.42	8.11	0.9	0.19		1.81	0.31	3.29	49.36667
16-04-2013	Far	D	LC	4.1	21.86	47.59	31.03	6.92	8.08	0.9	0.25		0.83	0	2.25	91.2
16-04-2013	Far	D	LC	4.1	21.86	47.59	31.03	6.92	8.08	0.9	0.23		1.28	0.26	7.03	144.9
16-04-2013	Far	E	LC	0.1	22.54	48.05	31.35	7.16	8.11	0.5	0.18		0.58	0.29	4.08	140.8
16-04-2013	Far	E	LC	0.1	22.54	48.05	31.35	7.16	8.11	0.5	0.19		3.12	1.02	4.32	36.72
16-04-2013	Far	E	LC	3.4	22.38	48.04	31.35	7.14	8.1	0.5	0.19		1.63	0.28	4.15	42.68
16-04-2013	Far	E	LC	3.4	22.38	48.04	31.35	7.14	8.1	0.5	0.2	16.16	1.27	0.34	4.48	33.92
16-04-2013	Near	F	LC	0.1	23.45	47.09	30.65	7.4	8.15	0.5	0.45	15.47	1.35	1.78	6.56	28.63333
16-04-2013	Near	F	LC	0.1	23.45	47.09	30.65	7.4	8.15	0.5	0.44	15.47	1.98	0.73	7.94	32.2
16-04-2013	Near	F	LC	1.6	23.38	47.09	30.65	7.32	8.15	0.5	0.44	15.42	1.34	0	12.51	33.16667
16-04-2013	Near	F	LC	1.6	23.38	47.09	30.65	7.32	8.15	0.5	0.43	15.64	1.6		9.15	31.5
16-04-2013	Near	FD	LC	0.1	24.04	45.77	29.68	7.39	8.1	0.5					7.29	33.96667
16-04-2013	Near	FD	LC	0.1	24.04	45.77	29.68	7.39	8.1	0.5					8.11	33.2
16-04-2013	Near	FD	LC	1.1	24.05	45.8	29.7	7.36	8.09	0.5					6.48	30.06667
16-04-2013	Near	FD	LC	1.1	24.05	45.8	29.7	7.36	8.09	0.5					8.05	49.73333
02-10-2013	Far	15	LC	0.1	28.36	48.64	31.65	7.4	8.24	0.4	0.93	42.12	0.63	3	11.65	84.57143
02-10-2013	Far	15	LC	0.1	28.36	48.64	31.65	7.4	8.24	0.4	0.95	41.21	0.64	1.15	14.36	81.97143
02-10-2013	Far	15	LC	0.6	28.35	49.34	32.17	7.23	8.24	0.4	0.96	38.36	1.05	0.35	10.49	47.66667
02-10-2013	Far	15	LC	0.6	28.35	49.34	32.17	7.23	8.24	0.4	0.98	42.22	0.65	0.81	17.51	40.9
02-10-2013	Far	8	LC	0.1	28.28	51.64	33.86	6.35	8.22	1	0.69	41.03	0.3	4.5	9.25	16.25714
02-10-2013	Far	8	LC	0.1	28.28	51.64	33.86	6.35	8.22	1	0.72	41.36	1.03	0.7	7.67	12.37143
02-10-2013	Far	8	LC	2.7	28.15	51.65	33.86	6.36	8.23	1	0.77	45.48	0.56	1.08	16.6	16.42857
02-10-2013	Far	8	LC	2.7	28.15	51.65	33.86	6.36	8.23	1	0.75	40.99	0.67	0.33	12.95	23.02857
02-10-2013	Near	A	LC	0.1	28.88	46.97	30.42	6.88	8.14	0.5	0.73	95.83	4.03	1.91	18.52	48.9
02-10-2013	Near	A	LC	0.1	28.88	46.97	30.42	6.88	8.14	0.5	0.75	88.02	3.69	1.75	16.79	45.5
02-10-2013	Near	A	LC	1.4	28.89	46.82	30.43	6.89	8.13	0.5	0.73	88.42	3.61	1.74	16.79	56.43333
02-10-2013	Near	A	LC	1.4	28.89	46.82	30.43	6.89	8.13	0.5	0.68	93.53	2.55	1.34	11.38	92.93333
02-10-2013	Near	B	LC	0.1	28.53	48.01	31.19	7.77	8.21	0.6	0.21	73.62	1.76	1.09	16.87	24.93333
02-10-2013	Near	B	LC	0.1	28.53	48.01	31.19	7.77	8.21	0.6	0.21	72.48	0.82	0.64	15.78	26.23333
02-10-2013	Near	B	LC	2	27.85	48.24	31.48	6.73	8.15	0.6	0.32	74.22	2.46	0.63	14.92	42.53333
02-10-2013	Near	B	LC	2	27.85	48.24	31.48	6.73	8.15	0.6	0.31	73.66	2.44	0.64	15.67	40.8
02-10-2013	Far	C	LC	0.1	27.73	47.26	30.67	6.19	8.08	0.7	0.26	25.41	1.01	1.84	6.98	27.71429
02-10-2013	Far	C	LC	0.1	27.73	47.26	30.67	6.19	8.08	0.7	0.26	25.73	0.97	0.63	6.45	23.42857
02-10-2013	Far	C	LC	3	27.64	47.33	30.72	5.93	8.11	0.7	0.29	24.9	1.51	1.18	7.69	24.71429
02-10-2013	Far	C	LC	3	27.64	47.33	30.72	5.93	8.11	0.7	0.28	24.39	1.52	1.18	6.8	23.05714
02-10-2013	Far	D	LC	0.1	28.03	44.41	28.6	6.63	8.23	1.2	0.29	26	1.4	0.3	3.77	22.08571
02-10-2013	Far	D	LC	0.1	28.03	44.41	28.6	6.63	8.23	1.2	0.31	28.56	0.91	0.52	5.42	19.71429
02-10-2013	Far	D	LC	4.1	27.89	46.78	30.32	5.84	8.21	1.2	0.47	31.23	1.54	0	6.38	45.53333
02-10-2013	Far	D	LC	4.1	27.89	46.78	30.32	5.84	8.21	1.2	0.47	32.46	2.98	0.42	6.55	42.73333
02-10-2013	Far	E	LC	0.1	28.27	48.95	31.89	6.48	8.22	0.5	0.6	40.03	1.01	3.11	8.55	29.48571
02-10-2013	Far	E	LC	0.1	28.27	48.95	31.89	6.48	8.22	0.5	0.62	39.44	1.44	0.86	6.39	28.8
02-10-2013	Far	E	LC	3.5	28.08	49.11	32	6.4	8.21	0.5	0.68	37.54	2.75	0.57	9.16	55.03333
02-10-2013	Far	E	LC	3.5	28.08	49.11	32	6.4	8.21	0.5	0.69	40.33	1.83	0.45	9.85	48.86667
02-10-2013	Near	F	LC	0.1	28.03	51.52	33.78	6.99	8.22	0.6	0.75	46.42	0.78	1.71	11.31	26.74286
02-10-2013	Near	F	LC	0.1	28.03	51.52	33.78	6.99	8.22	0.6	0.75	44.15	0.55	0.31	9.84	25.17143
02-10-2013	Near	F	LC	1.5	27.69	51.64	33.86	6.27	8.22	0.6	0.74	43.82	2.21	1.75	12.33	54.5
02-10-2013	Near	F	LC	1.5	27.69	51.64	33.86	6.27	8.22	0.6	0.73	42.74	0	0.17	13.15	49.23333
02-10-2013	Near	FD	LC	0.1	29.18	46.64	30.09	7.96	8.15	0.5	3.4	117.06	2.83	4.9	14.4	36.37143
02-10-2013	Near	FD	LC	0.1	29.18	46.64	30.09	7.96	8.15	0.5	3.55	121.56	1.55	4.83	13.16	40.42857
02-10-2013	Near	FD	LC	1.1	29.07	47.3	30.66	7.97	8.16	0.5	0.55	95.91	0.65	1.05	18.51	73.74286
02-10-2013	Near	FD	LC	1.1	29.07	47.3	30.66	7.97	8.16	0.5	0.54	104.08	0.38	0.62	24.31	75.6
08-04-2011	Near	A	NC	0.1	22.98	50.2	32.92	6.64	8.01	0.2	1.74	92.89	1.14	0.7	9.39	339.0222
08-04-2011	Near	A	NC	0.1	22.98	50.2	32.92	6.64	8.01	0.2	1.84	95.82	1.08	0.6	11.13	298.4
08-04-2011	Near	A	NC	1.2	22.98	50.19	32.91	6.61	8.01	0.2	1.8	94.11	0.98	0.4	5.9	NA
08-04-2011	Near	A	NC	1.2	22.98	50.19	32.91	6.61	8.01	0.2	1.85	94.3	1.02	0.54	13.57	NA
08-04-2011	Near	B	NC	0.1	22.93	50.03	32.8	6.66	8.07	0.4	0.57	63.1	1.16	0.56	7.58	171.1
08-04-2011	Near	B	NC	0.1	22.93	50.03	32.8	6.66	8.07	0.4	0.6	62.03	0.66	0.41	10.94	188.74
08-04-2011	Near	B	NC	1.1	22.93	50	32.8	6.64	8.07	0.4	0.64	64.82	0.31	0.3	14.94	NA
08-04-2011	Near	B	NC	1.1	22.93	50	32.8	6.64	8.07	0.4	0.58	60.68	0	0	13.99	NA
08-04-2011	Far	C	NC	0.1	22.61	48.79	31.9	6.84	8.03	0.4	0.37	24.06	0.86	0.37	4.98	94.9
08-04-2011	Far	C	NC	0.1	22.61	48.79	31.9	6.84	8.03	0.4	0.38	24.22	0.92	1.11	5.66	95.2
08-04-2011	Far	C	NC	3.9	22.61	48.85	31.94	6.82	8.05	0.4	0.34	24.04	0.76	0.61	6.74	NA
08-04-2011	Far	C	NC	3.9	22.61	48.85	31.94	6.82	8.05	0.4	0.34	23.93	0.93	1.07	6.21	NA
08-04-2011	Far	D	NC	0.1	23	49.72	32.57	7.01	8.17	0.6	0.21	16.37	0.73	0.49	2.59	24.22
08-04-2011	Far	D	NC	0.1	23	49.72	32.57	7.01	8.17	0.6	0.21	17.19	0.58	0.69	4.74	28.72
08-04-2011	Far	D	NC	3.3	22.91	49.67	32.53	6.88	8.16	0.6	0.21	16.87	1.33	1.05	4.3	NA
08-04-2011	Far	D	NC	3.3	22.91	49.67	32.53	6.88	8.16	0.6	0.21	17.08	1.19	1.35	3.82	NA
08-04-2011	Far	E	NC	0.1	23.21	49.51	32.41	6.99	8.17	0.5	0.2	16.97	0.65	0.25	6.78	37.58
08-04-2011	Far	E	NC	0.1	23.21	49.51	32.41	6.99	8.17	0.5	0.2	18.94	1.08	0	3.49	28.92
08-04-2011	Far	E	NC	3	23.2	49.5	32.41	6.96	8.17	0.5	0.2	16.53	0.83	0	5.08	NA
08-04-2011	Far	E	NC	3	23.2	49.5	32.41	6.96	8.17	0.5	0.19	15.84	0.64	0	6.83	NA
14-07-2011	Near	A	NC	0.1	30.08	59.46	39.64	6.35	8.02	0.3	1.83	88.48	0	1.43	9.71	91.12
14-07-2011	Near	A	NC	0.1	30.08	59.46	39.64	6.35	8.02	0.3	1.81	92.47	0	1.36	8.83	94.8
14-07-2011	Near	A	NC	1.2	30.1	59.42	39.62	6.33	8.02	0.3	1.84	91.82	0	0.49	8.12	NA
14-07-2011	Near	A	NC	1.2	30.1	59.42	39.62	6.33	8.02	0.3	1.81	90.08	0	0.53	10.11	NA
14-07-2011	Near	B	NC	0.1	30.78	56.83	37.63	6.13	8.16	0.5	0.54	51.85	0	1.75	8.36	95.28
14-07-2011	Near	B	NC	0.1	30.78	56.83	37.63	6.13	8.16	0.5	0.53	52.95	0	0.43	8.22	87.84
14-07-2011	Near	B	NC	1.2	30.8	56.81	37.62	6.02	8.17	0.5	0.54	50.95	0	0.42	9.78	NA
14-07-2011	Near	B	NC	1.2	30.8	56.81	37.62	6.02	8.17	0.5	0.56	52.07	0	0.37	8.98	NA
14-07-2011	Far	C	NC	0.1	30.22	55.88	36.93	6.18	8.2	0.8	0.24	34.12	0.36	1.4	6.08	34.86667
14-07-2011	Far	C	NC	0.1	30.22	55.88	36.93	6.18	8.2	0.8	0.27	35.51	0	0.56	4.43	34.06667
14-07-2011	Far	C	NC	3.8	29.94	56.94	37.74	4.58	8.08	0.8	0.25	34.61	0	0.79	4.44	NA
14-07-2011	Far	C	NC	3.8	29.94	56.94	37.74	4.58	8.08	0.8	0.24	35.85	0	0	8.74	NA
14-07-2011	Far	D	NC	0.1	30.24	59.13	39.38	5.88	8.27	0.4	0.24	50.67	0	0.87	6.93	78.66667
14-07-2011	Far	D	NC	0.1	30.24	59.13	39.38	5.88	8.27	0.4	0.24	48.05	0	0.7	8.47	35.7
14-07-2011	Far	D	NC	3.4	30.11	59.07	39.34	5.27	8.25	0.4	0.25	44.44	0	0.79	7.43	NA
14-07-2011	Far	D	NC	3.4	30.11	59.07	39.34	5.27	8.25	0.4	0.24	48.56	0	0.51	12.29	NA
14-07-2011	Far	E	NC	0.1	30.03	56.77	37.62	6.15	8.21	1	0.2	37.9	0.25	0.81	5.74	26.43333
14-07-2011	Far	E	NC	0.1	30.03	56.77	37.62	6.15	8.21	1	0.46	37.63	0	1.08	5.35	23.8
14-07-2011	Far	E	NC	2.8	29.92	57.56	38.21	5.41	8.23	1	0.22	38.12	0	0.31	7.67	NA
14-07-2011	Far	E	NC	2.8	29.92	57.56	38.21	5.41	8.23	1	0.22	38.81	0	0.38	5.09	NA
07-10-2011	Near	A	NC	0.1	26.18	66.47	45.17	5.98	8.06	0.4	1.31	96.09	0	0	13.89	60.16
07-10-2011	Near	A	NC	0.1	26.18	66.47	45.17	5.98	8.06	0.4	1.2	88.56	0	0	11.39	72.84
07-10-2011	Near	A	NC	1.5	26.18	66.46	45.14	5.95	8.05	0.4	1.37	97.15	0	0	9.45	NA
07-10-2011	Near	A	NC	1.5	26.18	66.46	45.14	5.95	8.05	0.4	1.4	101.54	0	0	10.38	NA
07-10-2011	Near	B	NC	0.1	26.57	63.86	43.12	6.08	8.05	0.5	0.85	62.15	0	0.44	6.71	54.76
07-10-2011	Near	B	NC	0.1	26.57	63.86	43.12	6.08	8.05	0.5	0.84	63.99	0	0.52	4.77	55.36
07-10-2011	Near	B	NC	1.2	26.57	63.81	43.08	6.06	8.05	0.5	0.79	63.55	0	0	6.19	NA
07-10-2011	Near	B	NC	1.2	26.57	63.81	43.08	6.06	8.05	0.5	0.84	64.61	0	0	7.31	NA
07-10-2011	Far	C	NC	0.1	26.53	61.7	41.45	6.27	8.12	0.7	0.32	38.63	0	0.52	4.71	46.86667
07-10-2011	Far	C	NC	0.1	26.53	61.7	41.45	6.27	8.12	0.7	0.32	38.76	0	0	4.18	38.6
07-10-2011	Far	C	NC	4	26.51	61.77	41.5	6.21	8.11	0.7	0.34	40.04	0	0	4.72	NA
07-10-2011	Far	C	NC	4	26.51	61.77	41.5	6.21	8.11	0.7	0.34	41.66	0	0	4.77	NA
07-10-2011	Far	D	NC	0.1	26.66	64.33	43.47	6.21	8.11	0.9	0.39	60.03	0	0.28	7.7	44.1
07-10-2011	Far	D	NC	0.1	26.66	64.33	43.47	6.21	8.11	0.9	0.39	59.34	0	0.47	6.25	32.13333
07-10-2011	Far	D	NC	3.3	26.47	64.24	43.41	5.91	8.09	0.9	0.34	50.91	0	0	3.68	NA
07-10-2011	Far	D	NC	3.3	26.47	64.24	43.41	5.91	8.09	0.9	0.38	58.66	0	0.26	2.96	NA
07-10-2011	Far	E	NC	0.1	27.35	60.26	40.33	6.31	8.12	0.7	0.3	33.52	0	0	4.01	30.76667
07-10-2011	Far	E	NC	0.1	27.35	60.26	40.33	6.31	8.12	0.7	0.33	35.28	0	0	4.69	33.4
07-10-2011	Far	E	NC	3	26.58	61.54	41.34	5.11	8.09	0.7	0.45	45.17	0.7	0	7.13	NA
07-10-2011	Far	E	NC	3	26.58	61.54	41.34	5.11	8.09	0.7	0.45	43.93	0.72	0	7.12	NA
11-01-2012	Near	A	NC	0.1	14.74	60.93	40.89	8.61	8.12	1.1	0.85	0	0	0.85	0.35	19.25714
11-01-2012	Near	A	NC	0.1	14.74	60.93	40.89	8.61	8.12	1.1	0.89	0	0	0.33	0.3	16.65714
11-01-2012	Near	A	NC	0.8	14.74	60.92	40.88	8.72	8.12	1.1	0.84	0	0	0	0.45	NA
11-01-2012	Near	A	NC	0.8	14.74	60.92	40.88	8.72	8.12	1.1	0.89	0	0	0.37	0.34	NA
11-01-2012	Near	B	NC	0.1	15.29	56.88	37.86	7.94	8.16	1	0.23	11.41	0	0.34	0.88	17.08571
11-01-2012	Near	B	NC	0.1	15.29	56.88	37.86	7.94	8.16	1	0.23	10.51	0	0.31	0.97	14.08571
11-01-2012	Near	B	NC	0.7	15.3	56.86	37.84	7.95	8.17	1	0.24	10.54	0	0	1.06	NA
11-01-2012	Near	B	NC	0.7	15.3	56.86	37.84	7.95	8.17	1	0.25	10.7	0	0.48	0.62	NA
11-01-2012	Far	C	NC	0.1	15.64	54.31	35.96	8.25	8.1	2.5	0.16	13.15	0	0.67	0.99	40.66667
11-01-2012	Far	C	NC	0.1	15.64	54.31	35.96	8.25	8.1	2.5	0.16	13.21	0	0	1.28	31.2
11-01-2012	Far	C	NC	3.4	15.87	56.48	37.76	8.03	8.13	2.5	0.15	12.75	0	0	1.5	NA
11-01-2012	Far	C	NC	3.4	15.87	56.48	37.76	8.03	8.13	2.5	0.17	11.74	0	0.1	1.45	NA
11-01-2012	Far	D	NC	0.1	14.51	54.65	36.17	8.14	8.04	1	0	19.61	0	0.31	2.66	24.03333
11-01-2012	Far	D	NC	0.1	14.51	54.65	36.17	8.14	8.04	1	0	19.4	0	0	1.28	22.56667
11-01-2012	Far	D	NC	2.5	14.31	54.63	36.15	8.11	8.04	1	0	18.93	0	0	2.65	NA
11-01-2012	Far	D	NC	2.5	14.31	54.63	36.15	8.11	8.04	1	0	19.41	0	0	1.44	NA
11-01-2012	Far	E	NC	0.1	15.23	54.01	35.72	7.93	8	0.8	0.15	16.36	0	0.79	2.25	27.93333
11-01-2012	Far	E	NC	0.1	15.23	54.01	35.72	7.93	8	0.8	0.15	16.3	0	0.32	2.07	29.73333
11-01-2012	Far	E	NC	2.5	15.17	54.01	35.73	7.92	8.02	0.8	0.16	16.76	0	0	2.18	NA
11-01-2012	Far	E	NC	2.5	15.17	54.01	35.73	7.92	8.02	0.8	0.16	16.43	0	0	1.89	NA
13-04-2012	Near	A	NC	0.1	24.18	53.81	35.57	6.31	7.85	0.2	0.98	99.75	5.23	1.28	7.43	268.4
13-04-2012	Near	A	NC	0.1	24.18	53.81	35.57	6.31	7.85	0.2	1.01	85.47	5.85	0.87	8.82	228.6
13-04-2012	Near	A	NC	1.2	24.18	53.82	35.57	6.32	7.85	0.2	1.09	98.27	5.12	0.85	5.93	NA
13-04-2012	Near	A	NC	1.2	24.18	53.82	35.57	6.32	7.85	0.2	1.07	92.64	5.06	1.09	7.59	NA
13-04-2012	Near	B	NC	0.1	24.55	52.91	34.89	6.22	7.94	0.3	0.71	76.51	4.42	0.52	8.65	226.3333
13-04-2012	Near	B	NC	0.1	24.55	52.91	34.89	6.22	7.94	0.3	0.79	87.76	5.69	2.54	6.56	185.1333
13-04-2012	Near	B	NC	1.1	24.57	52.87	34.87	6.17	7.94	0.3	0.75	71.03	4.31	1.82	5.52	NA
13-04-2012	Near	B	NC	1.1	24.57	52.87	34.87	6.17	7.94	0.3	0.82	67.9	5.48	2.63	5.71	NA
13-04-2012	Far	C	NC	0.1	24.86	49.28	32.21	6.57	8.02	0.4	0	26.44	1.18	0	5.72	123.3333
13-04-2012	Far	C	NC	0.1	24.86	49.28	32.21	6.57	8.02	0.4	0.11	12.77	0.81	0.08	4.91	129.8667
13-04-2012	Far	C	NC	3.9	24.87	49.27	32.21	6.47	8.03	0.4	0.11	24.15	1.24	0	5.54	NA
13-04-2012	Far	C	NC	3.9	24.87	49.27	32.21	6.47	8.03	0.4	0.13	24.01	0.94	0	3.77	NA
13-04-2012	Far	D	NC	0.1	25.24	56.97	37.91	6.96	8.17	0.6	0	45.98	1.03	0.27	17.36	33.32
13-04-2012	Far	D	NC	0.1	25.24	56.97	37.91	6.96	8.17	0.6	0	50.97	0.58	0.27	14.9	55.84
13-04-2012	Far	D	NC	3.3	25.17	56.97	37.92	6.84	8.17	0.6	0	60.59	0	0	19.88	NA
13-04-2012	Far	D	NC	3.3	25.17	56.97	37.92	6.84	8.17	0.6	0	51.43	0	0	13.98	NA
13-04-2012	Far	E	NC	0.1	25.36	50.93	33.42	7.23	8.15	0.6	0	26.36	1.23	0	14.98	17.16667
13-04-2012	Far	E	NC	0.1	25.36	50.93	33.42	7.23	8.15	0.6	0	21.18	0.71	0	5.81	21.43333
13-04-2012	Far	E	NC	3	25.15	51.62	34.11	6.96	8.14	0.6	0	27.24	0.44	0	9.66	NA
13-04-2012	Far	E	NC	3	25.15	51.62	34.11	6.96	8.14	0.6	0	34.28	0.98	0	9.65	NA
09-07-2012	Near	A	NC	0.1	30.12	47.15	30.52	6.99	8.21	0.7	2.16	164.42	0.84	1.11	6.6	24.9
09-07-2012	Near	A	NC	0.1	30.12	47.15	30.52	6.99	8.21	0.7	2.16	157.9	0.63	0.53	4.9	20.43333
09-07-2012	Near	A	NC	1	29.59	49.13	31.97	5.5	8.07	0.7	1.98	131.05	0.84	0.45	7.24	NA
09-07-2012	Near	A	NC	1	29.59	49.13	31.97	5.5	8.07	0.7	1.97	142.51	1.04	0.46	5.16	NA
09-07-2012	Near	B	NC	0.1	29.96	51.14	33.43	5.92	8.12	0.6	1.32	129.65	0.52	0.8	6.58	41.68
09-07-2012	Near	B	NC	0.1	29.96	51.14	33.43	5.92	8.12	0.6	1.37	122.32	0.86	0.43	6.83	40.8
09-07-2012	Near	B	NC	1.1	29.88	51.59	33.79	5.54	8.08	0.6	1.36	122.52	1.32	0.38	6.03	NA
09-07-2012	Near	B	NC	1.1	29.88	51.59	33.79	5.54	8.08	0.6	0.56	117.19	1.03	0.4	4.58	NA
09-07-2012	Far	C	NC	0.1	30.23	55.16	36.4	6.24	8.26	1	0.31	63.2	0.45	3.11	3.3	24.46667
09-07-2012	Far	C	NC	0.1	30.23	55.16	36.4	6.24	8.26	1	0.3	61.39	0.46	0.48	3.72	26.36667
09-07-2012	Far	C	NC	3	30.15	55.92	36.98	6.02	8.28	1	0.11	46.55	0.51	0.27	3.56	NA
09-07-2012	Far	C	NC	3	30.15	55.92	36.98	6.02	8.28	1	0.11	46.63	0.41	0.42	3.2	NA
09-07-2012	Far	D	NC	0.1	29.76	59.71	39.84	5.6	8.44	1.1	0	64.78	0	0.87	12.22	15.25714
09-07-2012	Far	D	NC	0.1	29.76	59.71	39.84	5.6	8.44	1.1	0	64.89	0	0.25	6.88	17.82857
09-07-2012	Far	D	NC	2.5	29.45	60	40.07	5.6	8.45	1.1	0	63.78	0	0	11.2	NA
09-07-2012	Far	D	NC	2.5	29.45	60	40.07	5.6	8.45	1.1	0	64.66	0	0	8	NA
09-07-2012	Far	E	NC	0.1	29.94	58.82	39.16	5.58	8.37	0.8	0	63.88	0.38	0.6	6.62	27.4
09-07-2012	Far	E	NC	0.1	29.94	58.82	39.16	5.58	8.37	0.8	0	60.69	0.34	0.36	7.16	28.02857
09-07-2012	Far	E	NC	2.8	29.82	58.93	39.25	5.22	8.36	0.8	0.1	64.86	0.34	0	8.05	NA
09-07-2012	Far	E	NC	2.8	29.82	58.93	39.25	5.22	8.36	0.8	0.11	63.93	0.36	0	4.96	NA
01-10-2012	Near	A	NC	0.1	27.38	62.27	41.94	7.85	8.06	0.5	1.91	141.12	1.3	0.52	23.34	22.86667
01-10-2012	Near	A	NC	0.1	27.38	62.27	41.94	7.85	8.06	0.5	2.87	149.81	1.58	0.72	30.73	30.83333
01-10-2012	Near	A	NC	0.9	25.24	62.41	42.03	7.75	8.04	0.5	1.81	121.42	0.52	0	25.66	NA
01-10-2012	Near	A	NC	0.9	25.24	62.41	42.03	7.75	8.04	0.5	1.82	120.21	1.05	0	26.81	NA
01-10-2012	Near	B	NC	0.1	27.44	61.69	41.43	7.47	8.12	0.4	1.3	106.9	0	0	10.99	37.16667
01-10-2012	Near	B	NC	0.1	27.44	61.69	41.43	7.47	8.12	0.4	1.48	120.16	0.26	0.91	15.25	45.83333
01-10-2012	Near	B	NC	0.9	25.47	61.44	41.3	6.62	8.09	0.4	1.44	114.53	0	0	20.5	NA
01-10-2012	Near	B	NC	0.9	25.47	61.44	41.3	6.62	8.09	0.4	1.48	115.63	0	0	14.58	NA
01-10-2012	Far	C	NC	0.1	27.47	60.62	40.65	6.9	8.2	0.9	0.25	45.21	0.87	0	4.95	18.57143
01-10-2012	Far	C	NC	0.1	27.47	60.62	40.65	6.9	8.2	0.9	0.23	46.38	1.37	0.49	8.87	20.45714
01-10-2012	Far	C	NC	3.7	26.41	60.86	40.83	5.82	8.18	0.9	0.33	46.66	1.34	0.49	6.11	NA
01-10-2012	Far	C	NC	3.7	26.41	60.86	40.83	5.82	8.18	0.9	0.33	47.63	1.64	0.43	10.62	NA
01-10-2012	Far	D	NC	0.1	25.8	63.07	42.54	6.66	8.29	1	0	37.88	0.32	2.39	11.64	16.68571
01-10-2012	Far	D	NC	0.1	25.8	63.07	42.54	6.66	8.29	1	0	37.33	0	0.95	9.62	16.37143
01-10-2012	Far	D	NC	2.9	25.35	63.07	42.56	6.6	8.3	1	0	37.48	0	0	12.39	NA
01-10-2012	Far	D	NC	2.9	25.35	63.07	42.56	6.6	8.3	1	0	38.59	0	0	10.44	NA
01-10-2012	Far	E	NC	0.1	25.92	61.98	41.69	6.47	8.24	0.9	0.15	38.52	0.3	0	12.17	16.6
01-10-2012	Far	E	NC	0.1	25.92	61.98	41.69	6.47	8.24	0.9	0.22	38.97	1.15	1	10.33	15.5
01-10-2012	Far	E	NC	2.8	25.88	62.14	41.82	6.27	8.24	0.9	0.19	38.45	0	0	11.32	NA
01-10-2012	Far	E	NC	2.8	25.88	62.14	41.82	6.27	8.24	0.9	0.18	38.36	0	0	10.3	NA
07-01-2013	Near	A	NC	0.1	11.56	60.15	40.1	10.1	8.11	0.6	1.44	68.87	2.1	3.13	22.24	33.56667
07-01-2013	Near	A	NC	0.1	11.56	60.15	40.1	10.1	8.11	0.6	1.44	67.87	2.25	3.48	16.93	36.63333
07-01-2013	Near	A	NC	1.3	11.49	60.19	40.15	10.07	8.11	0.6	1.47	68.51	2.19	3.38	17.86	44.68
07-01-2013	Near	A	NC	1.3	11.49	60.19	40.15	10.07	8.11	0.6	1.47	68.51	2.19	3.38	17.86	50.32
07-01-2013	Near	B	NC	0.1	11.23	57.57	38.18	8.9	8.08	0.7	0.56	34.45	1.05	0.8	1.35	29.2
07-01-2013	Near	B	NC	0.1	11.23	57.57	38.18	8.9	8.08	0.7	0.58	33.75	1.21	1.38	0.84	31.43333
07-01-2013	Near	B	NC	1.2	11.23	57.57	38.18	8.9	8.08	0.7	0.57	33.78	1.18	1.35	0.84	30.92
07-01-2013	Near	B	NC	1.2	11.19	57.57	38.18	8.91	8.08	0.7	0.58	32.81	2.01	0.75	1.62	42.96
07-01-2013	Far	C	NC	0.1	11.53	56.64	37.51	9.14	8.13	1.4	0.22	22.98	4.22	1.3	4.04	26.83333
07-01-2013	Far	C	NC	0.1	11.53	56.64	37.51	9.14	8.13	1.4	0.22	22.69	0.97	0.86	3.09	27.43333
07-01-2013	Far	C	NC	4	11.53	56.64	37.51	9.14	8.13	1.4	0.22	22.49	0.97	0.86	3.09	24
07-01-2013	Far	C	NC	4	10.91	57.55	38.14	8.96	8.12	1.4	0.44	30.87	2.56	0.4	2.02	23.46667
07-01-2013	Far	D	NC	0.1	11.64	54.68	36.03	9.26	8.13	1.4	0.18	17.77	2.28	1.07	3.57	22.66667
07-01-2013	Far	D	NC	0.1	11.64	54.68	36.03	9.26	8.13	1.4	0.17	17.87	1.67	0.34	5.31	21.06667
07-01-2013	Far	D	NC	2.7	10.93	54.89	36.17	9.31	8.13	1.4	0.18	19.4	2.9	0.46	3.67	21.06667
07-01-2013	Far	D	NC	2.7	10.93	54.89	36.17	9.31	8.13	1.4	0.2	18.71	3.19	0.27	3.79	21.06667
07-01-2013	Far	E	NC	0.1	11.38	53.82	35.42	9.11	8.11	1.8	0.24	17.34	1.5	0.48	3.75	19.43333
07-01-2013	Far	E	NC	0.1	11.38	53.82	35.42	9.11	8.11	1.8	0.25	17.89	1.9	2.19	2.56	18.56667
07-01-2013	Far	E	NC	3	11.39	54.29	35.78	9.13	8.1	1.8	0.22	19.58	1.37	0.29	4.79	24.33333
07-01-2013	Far	E	NC	3	11.39	54.29	35.78	9.13	8.1	1.8	0.22	20.08	2.55	1.15	2.71	22.03333
05-04-2013	Near	A	NC	0.1	20.04	59.56	39.94	7.74	7.98	0.4	1	69.61	2.2		6.77	49.06667
05-04-2013	Near	A	NC	0.1	20.04	59.56	39.94	7.74	7.98	0.4	0.99	70.18	1.95	4.07	6.13	69.7
05-04-2013	Near	A	NC	1	19.9	59.59	39.95	7.74	7.98	0.4	1.08	68.52	2.24	3.29	5.69	73.7
05-04-2013	Near	A	NC	1	19.9	59.59	39.95	7.74	7.98	0.4	1.04	69.4	3.07	3.23	4.48	72.96667
05-04-2013	Near	B	NC	0.1	20.27	56.6	37.72	7.49	8.02	0.4	0.53	42.03	0.98	9.59	4.84	52.46667
05-04-2013	Near	B	NC	0.1	20.27	56.6	37.72	7.49	8.02	0.4	0.54	43.97	0.67	0.75	6.38	49.5
05-04-2013	Near	B	NC	0.9	19.9	56.97	38.1	7.48	8.01	0.4	0.51	43.9		3.46	7.52	56.32
05-04-2013	Near	B	NC	0.9	19.9	56.97	38.1	7.48	8.01	0.4	0.53	44.06	0	0.61	8.49	56.32
05-04-2013	Far	C	NC	0.1	20.61	53.12	35.11	7.53	8.11	0.8	0	12.24	0	0	3.11	19.1
05-04-2013	Far	C	NC	0.1	20.61	53.12	35.11	7.53	8.11	0.8	0.1	12.38	0.88	0.4	1.45	18.43333
05-04-2013	Far	C	NC	3.7	18.73	54.99	36.5	7.08	8.09	0.8	0.21	28.62	0	0	2.57	35.76
05-04-2013	Far	C	NC	3.7	18.73	54.99	36.5	7.08	8.09	0.8	0.22	29.04	0	0	3.44	36.56
05-04-2013	Far	D	NC	0.1	18.96	50.68	33.31	8	8.19	1.3	0	0	1.05	1.25	1.97	11.28571
05-04-2013	Far	D	NC	0.1	18.96	50.68	33.31	8	8.19	1.3	0	0	0.99	0.67	2.77	10.08571
05-04-2013	Far	D	NC	2.8	18.73	51.39	33.84	7.72	8.19	1.3	0	0	0.51	0	3.24	35.93333
05-04-2013	Far	D	NC	2.8	18.73	51.39	33.84	7.72	8.19	1.3	0		1.01	0	3.13	21.2
05-04-2013	Far	E	NC	0.1	19.69	50.23	32.99	7.82	8.13	0.9	0	0	1.63	1.05	3.42	20.37143
05-04-2013	Far	E	NC	0.1	19.69	50.23	32.99	7.82	8.13	0.9	0.1	0	1.13	0.31	2.36	16.62857
05-04-2013	Far	E	NC	2.9	19.68	50.47	33.8	7.65	8.14	0.9	0.27	27.11	3.28	0	3.22	19.31429
05-04-2013	Far	E	NC	2.9	19.68	50.47	33.8	7.65	8.14	0.9	0.1	0	0.64	0.33	2.17	17.94286
08-10-2013	Near	A	NC	0.1	24.21	60.69	40.75	7.11	8.12	0.5	1.35	103.63	0.53	0.42	8.76	28.88571
08-10-2013	Near	A	NC	0.1	24.21	60.69	40.75	7.11	8.12	0.5	1.36	102.82	0.54	0.4	8.03	23.71429
08-10-2013	Near	A	NC	1.3	23.38	61.14	41.11	7.26	8.12	0.5	1.1	99.6	0.71	0.26	12.65	38.97143
08-10-2013	Near	A	NC	1.3	23.38	61.14	41.11	7.26	8.12	0.5	1.09	100.52	0.64	0.25	11.53	38.37143
08-10-2013	Near	B	NC	0.1	25.56	60.27	40.42	7.38	8.16	0.5	0.55	71.51	0.37	0.28	3.1	31.14286
08-10-2013	Near	B	NC	0.1	25.56	60.27	40.42	7.38	8.16	0.5	0.58	76.02	0.37	0.5	9.12	31.91429
08-10-2013	Near	B	NC	1.2	24.48	60.27	40.43	7.35	8.17	0.5	0.56	75.65	0.28	0	8.85	31.88571
08-10-2013	Near	B	NC	1.2	24.48	60.27	40.43	7.35	8.17	0.5	0.55	75.93	0.28	0	8.87	28.45714
08-10-2013	Far	C	NC	0.1	26.4	57.12	38.01	7.13	8.21	1.4	0.1	35.59	0	0	4.69	12.725
08-10-2013	Far	C	NC	0.1	26.4	57.12	38.01	7.13	8.21	1.4	0.12	37.51	0.65	0.39	4.05	13.65
08-10-2013	Far	C	NC	4	25.43	57.54	38.36	5.43	8.19	1.4	0.15	38.97	0.42	0	7.21	59.06667
08-10-2013	Far	C	NC	4	25.43	57.54	38.36	5.43	8.19	1.4	0.14	38.21	0.63	0	7.27	60.13333
08-10-2013	Far	D	NC	0.1	24.92	54.94	36.4	7.11	8.21	1.5	0	23.71	0.36	0	2.8	7.377778
08-10-2013	Far	D	NC	0.1	24.92	54.94	36.4	7.11	8.21	1.5	0	24.08	0.52	0.4	1.81	5.333333
08-10-2013	Far	D	NC	3.1	24.66	57.7	38.49	6.02	8.23	1.5	0	22.74			3.71	11.24444
08-10-2013	Far	D	NC	3.1	24.66	57.7	38.49	6.02	8.23	1.5	0	23.62	0.46	0	4.53	8.777778
08-10-2013	Far	E	NC	0.1	25.43	49.92	32.68	7.01	8.13	1.7	0.13	16.26	0.44	0	3.12	4.36
08-10-2013	Far	E	NC	0.1	25.43	49.92	32.68	7.01	8.13	1.7	0.14	17.51	0.58	0.61	3.05	6.88
08-10-2013	Far	E	NC	3.1	26.84	57.67	38.4	5.31	8.09	1.7	0.16	32.06	0.27	0.45	8.96	23.62857
08-10-2013	Far	E	NC	3.1	26.84	57.67	38.4	5.31	8.09	1.7	0.15	31.38	0.94	0	10.61	20.6

Est = Estuary.

NC = Nueces Estuary.

GE = Guadalupe Estuary.

LC = Lavaca-Colorado Estuary.

Dist = Distance, this is classified into “near” and “far” from the river inflow to primary and secondary estuary.

Abbreviation for water quality parameters.

Temp = Temperature in degree C.

DO = Dissolved oxygen in mg/L.

Sal = Salinty

Cond = Conductivity in μs/cm.

Chl = Chlorophyll-a in μg/L.

TSS = total suspended solids in mg/L.

PO4 = dissolved phosphate in μmol/L.

SiO4 = dissolved phosphate in μmol/L.

NH4 = dissolved ammonia in μmol/L.

NOx = dissolved nitrite + nitrate in μmol/L.

Total surface inflow from a river basin to an estuarine system was estimated from 2011 to 2013 by summing flows originating in gaged and ungagged watersheds (Table 2). Gaged flows were obtained from USGS streamflow records. Ungaged flows were the sum of three components: (1) computed streamflow, using a rainfall-runoff simulation model, based on precipitation over the watershed, here termed as “Modelled Flow” (2) flow diverted from streams by municipal, industrial, agricultural, and other users, here termed as “Diversion” and (3) unconsumed flow returned to streams, here termed as “Return Flow”. Thus, the inflow rates presented were the modeled inflow data where input to each estuary equals to the sum of gaged flow, modelled flow, and return flow, whereas output or amount of water exiting the system would be diversion plus evaporation (Table 2; https://waterdatafortexas.org/coastal/hydrology).

**Table 2:** Surface inflow data from 2011 to 2013 in the three Texas estuaries.

Estuaries	Month	Date	Gauge Flow (ac-ft/month)	Modelled Flow (ac-ft/month)	Diversion (ac-ft/month)	Return Flow (ac-ft/month)	Inflow (ac-ft/month)	Inflow (m3/month)
GE	1	January-11	93717	24155	7824.9	7139	117186.1	144546710.6
GE	2	February-11	64013	1216	3658.9	2391	63961.1	78894737.63
GE	3	March-11	58890	477	4363	2170.5	57174.5	70523602.26
GE	4	April-11	44599	128	3546.3	1674.9	42855.7	52861648.84
GE	5	May-11	37986	3816	3618	8487	46671	57567745.08
GE	6	June-11	33722	5629	4277.2	4086	39159.8	48302830.1
GE	7	July-11	22667	141	3538.1	2018.1	21288	26258322.24
GE	8	August-11	14744	157	3693	3597.3	14805.2	18261918.1
GE	9	September-11	16124	1559	3105	3691.4	18269.4	22534939.51
GE	10	October-11	43615	11130	3973.6	12299.9	63071.3	77797187.12
GE	11	November-11	30969	116	4565.1	2209.2	28729.1	35436770.27
GE	12	December-11	53487	344	4219.8	2441.5	52052.7	64205964.4
GE	1	January-12	124725	97	10452.1	3165.3	117535.2	144977318.5
GE	2	February-12	169342	6578	9171.6	6342.7	173091.1	213504410
GE	3	March-12	206246	13244	10137.2	7355.5	216708.3	267305353.9
GE	4	April-12	75240	16406	10988.9	7559.3	88216.5	108813288.4
GE	5	May-12	167238	9514	16886.8	10451.9	170317.1	210082736.5
GE	6	June-12	43100	1011	19644.3	4420.5	28887.1	35631660.11
GE	7	July-12	85607	56911	13813.8	9498.5	138202.7	170470266.4
GE	8	August-12	44498	2517	15915.2	2952.4	34052.2	42002707.66
GE	9	September-12	59592	37159	15452.8	23459.9	104758	129216897.8
GE	10	October-12	67635	16105	14857.3	2734.4	71617.1	88338260.51
GE	11	November-12	38138	1915	16095.5	1822	25779.5	31798497.66
GE	12	December-12	35262	2872	7797.8	1940	32276.1	39811923.83
GE	1	January-13	63419	13558	7057	11842.5	81762.4	100852285.2
GE	2	February-13	38843	12282	5664.7	8447.6	53907.9	66494316.49
GE	3	March-13	36571	236	8030	2346.8	31123.8	38390584.82
GE	4	April-13	45932	19841	10270.5	9125.9	64628.4	79717838.83
GE	5	May-13	126602	50114	11823	19345.7	184238.7	227254751.7
GE	6	June-13	61127	2080	15816	1885.5	49276.5	60781577.22
GE	7	July-13	31582	5790	19827.7	10893.8	28438.1	35077827.59
GE	8	August-13	17354	6399	15324.1	4207.9	12636.8	15587240.06
GE	9	September-13	27495	14987	9889.5	8245	40837.5	50372239.5
GE	10	October-13	92197	12999	8035	8496.9	105657.9	130326906.5
GE	11	November-13	152267	4882	7450.8	2822	152520.2	188130616.3
GE	12	December-13	45804	320	6818.4	1881	41186.6	50802847.37
LC	1	January-11	64305.3	57177	485	9815.8	130813.1	161355342.6
LC	2	February-11	36694.7	4307	382.2	5360.7	45980.2	56715657.1
LC	3	March-11	18909.6	2052	10435.6	1316.7	11842.7	14607733.6
LC	4	April-11	7324.5	845	3406	1196.1	5959.6	7351047.408
LC	5	May-11	15786.8	9305	1787.5	6092	29396.3	36259748.12
LC	6	June-11	16240.6	15860	1296.3	10667.8	41472.1	51155005.91
LC	7	July-11	7797.5	862	2807.3	1863.5	7715.7	9517161.636
LC	8	August-11	7069.1	970	694	1921.1	9266.2	11429672.38
LC	9	September-11	8190.7	4508	32.4	7266.5	19932.8	24586710.14
LC	10	October-11	20764.9	32324	1463.1	11475	63100.9	77833698.13
LC	11	November-11	17154.6	6705	1052.9	1124.3	23931	29518409.88
LC	12	December-11	50023	12423	5037.4	1249.9	58658.4	72353963.23
LC	1	January-12	145017.3	12118	21140.5	5922.6	141917.5	175052397.9
LC	2	February-12	184709.2	29716	60794.6	9665.9	163296.6	201423090.2
LC	3	March-12	300769.1	65867	52271.1	9851.8	324216.8	399914938.5
LC	4	April-12	66277.4	117570	21591.9	11200.3	173455.8	213954260.2
LC	5	May-12	87586.8	30818	9402.8	9794.4	118796.4	146532983.5
LC	6	June-12	10562.9	8197	4084.4	6067.4	20742.9	25585952.29
LC	7	July-12	187386.2	281270	2403.3	11093.6	477346.6	588797484.2
LC	8	August-12	15421.5	38008	2128.3	6278.2	57579.4	71023038.31
LC	9	September-12	28946.8	86572	1523.5	10667.4	124662.7	153768947.2
LC	10	October-12	38559.2	78173	6023.7	2566.3	113274.8	139722200.3
LC	11	November-12	18935.8	4474	1333.9	1095	23170.9	28580841.73
LC	12	December-12	20130.4	10813	880.4	6062.9	36125.8	44560451.78
LC	1	January-13	47518.9	28529	5198.8	10561.5	81410.6	100418346.9
LC	2	February-13	18532.1	24473	1311.4	8958.2	50651.8	62477982.26
LC	3	March-13	13722.1	898	1436.8	1268.8	14452.1	17826376.31
LC	4	April-13	47446.8	19939	10189.5	6447.2	63643.5	78502984.38
LC	5	May-13	33591.6	6041	6902.5	1663.6	34393.7	42423941.08
LC	6	June-13	13917.5	7753	1999.9	9647.4	29318	36163166.64
LC	7	July-13	15150.5	18593	1528	10079.1	42294.6	52169543.21
LC	8	August-13	7553	39723	1908.3	11307.8	56675.5	69908095.74
LC	9	September-13	18191.8	65550	1578.5	10716.4	92879.7	114565252.4
LC	10	October-13	89092.4	44425	42702.8	11005.9	101820.5	125593550.3
LC	11	November-13	263946.8	105108	69861.5	9465.3	308658.6	380724209.9
LC	12	December-13	50188	3034	15992.5	5983.1	43212.5	53301754.5
NC	1	January-11	4956	15657	3649.6	46903.6	63867.1	78778790.51
NC	2	February-11	3540	449	3238	20718.9	21469.8	26482568.9
NC	3	March-11	5392	181	4578	18958	19953	24611626.44
NC	4	April-11	6292	59	5514.5	18831.3	19667.9	24259961.29
NC	5	May-11	8068	5181	5573.6	33053.1	40728.6	50237913.53
NC	6	June-11	8268	181	6584.3	27717.8	29582.5	36489422.1
NC	7	July-11	6401	17	6928.1	29802.3	29292.2	36131342.86
NC	8	August-11	9677	108	8085.8	33406.9	35106	43302548.88
NC	9	September-11	11760	505	8050.2	33644.9	37859.8	46699306.1
NC	10	October-11	6891	922	7062.8	30818.6	31568.8	38939483.42
NC	11	November-11	12182	3	6081.1	18620.2	24724.1	30496682.87
NC	12	December-11	5569	40	5436.6	20548.5	20720.9	25558815.73
NC	1	January-12	5150	0	12511.8	20390	13028.3	16070147.48
NC	2	February-12	4308	14986	8915.4	24136.2	34514.8	42573315.5
NC	3	March-12	6043	491	10512.1	20766.4	16788.3	20708032.28
NC	4	April-12	7949	8429	11984.5	39596.1	43989.6	54260291.81
NC	5	May-12	10977	14491	10822.3	37179	51824.7	63924730.96
NC	6	June-12	28043	386	12957.5	38514.1	53985.6	66590157.89
NC	7	July-12	8317	243	11908.4	32303.8	28955.4	35715906.79
NC	8	August-12	11484	95	16256.4	34340.6	29663.2	36588963.94
NC	9	September-12	6365	4631	14480.8	36586.3	33101.5	40830038.22
NC	10	October-12	7144	2776	11791.5	34354.2	32482.7	40066760.8
NC	11	November-12	5169	44	12720.7	27295.2	19787.5	24407485.5
NC	12	December-12	4958	2	6232.8	29021.8	27749	34227836.52
NC	1	January-13	4423	284	5823	28442	27326	33706074.48
NC	2	February-13	3671	50	5092.9	18720	17348.1	21398534.39
NC	3	March-13	5300	0	6368.2	23118.5	22050.4	27198727.39
NC	4	April-13	5038	594	5802.6	22378.7	22208	27393123.84
NC	5	May-13	4997	986	5252.1	30993.9	31724.8	39131906.3
NC	6	June-13	7891	297	6547.4	35006.7	36647.3	45203711.6
NC	7	July-13	6287	6243	6002.7	39743.6	46270.9	57074229.73
NC	8	August-13	6651	722	6723.8	35188.2	35837.4	44204716.15
NC	9	September-13	6323	11236	5628	35100.2	47031.3	58012167.92
NC	10	October-13	19335	5469	5282.7	36200.2	55721.5	68731355.82
NC	11	November-13	18183	999	5375.3	35595.9	49402.6	60937119.05
NC	12	December-13	5010	138	5657	34467.7	33958.7	41887377.28

Downloaded date 01-01-2018.

Downloaded From https://waterdatafortexas.org/coastal/hydrology.

GE = Guadalupe Estuary.

NC = Nueces Estuary.

LC = Lavaca-Colorado Estuary.

The water quality data collected from the three estuaries was used to calculated difference between near and far stations in journal paper, i.e. reference 1 in the list. A weighted average value was calculated prior to running statistical analyses. SAS code used to calculate weighted average is provided here, MS word file names as “SAS code”.

## 2 Experimental design, materials, and methods

### 2.1 Experimental design

The estuaries have similar geographic structure but have different inflow regimes [2], [3], [4]. River inflow decreases from the north to the south; the average inflow for the Lavaca-Colorado, Guadalupe and Nueces Estuaries are 3679 × 10^6^m^3^yr^−1^, 2677 × 10^6^m^3^yr^−1^, and 348 × 10^6^m^3^yr^−1^ respectively [2]. The Nueces River discharges into the Nueces Estuary, the Guadalupe River discharges into the Guadalupe Estuary, and the Lavaca River and Colorado River discharges into the Lavaca-Colorado Estuary. In the three estuaries, 2012 had the highest river flow rates, followed by 2013, and 2011 had the lowest flow rates [1]. In this data we have used following gages: USGS 08211500 for NC, 08188810 for GE, and 08164800, 08164600, 08164000, 08164525, 08162600, and 08162000 for LC.

The stations were sampled quarterly from April 2011 to Oct 2013 in all three estuaries to collect water quality data listed in the attached data file. The three estuaries are shallow and well mixed, and the surface and bottom water quality values are similar. Stations in the Lavaca-Colorado, Guadalupe and Nueces Estuaries were located along salinity gradients from the major freshwater sources to the tidal inlets of the Gulf of Mexico. Stations A, B, and F were closer to the river mouth in the estuaries compared to stations C, D, and E, which were closer to the Gulf of Mexico (Table 1). The stations closer to freshwater sources are in the secondary bays and are called “near” treatment stations and those further from freshwater sources are in primary bays and are called “far” treatment stations. Samples were first average by date-estuary-treatment-station-depth, then averaged by date-estuary-treatment-station, and then average by date-estuary-treatment (see SAS code). This was necessary because there were many missing values and the number of stations in the treatments was unbalanced. The date-estuary-treatment-station data set was used for analysis of variance (ANOVA) where stations were replicates, and the average by date-estuary-treatment data set was used in the principal components analysis (PCA) (see reference [1]). The data was then transformed by the natural logarithm (ln) to help normalize the distribution of the residuals. ANOVA was performed using the GLM procedure [5] and all plots were created using SGPLOT, SGSCATTER and SAS ODS graphics designer [6]. SAS code used to obtain Fig 2 of reference [1] is provided here (Appendix A).

### 2.2 Water quality parameters

Dissolved oxygen (DO), pH, temperature, salinity and conductivity were measured at each sampling event using an YSI Hydro Sonde (YSI Model 556 MPS, Yellow Spring, OH, USA).

### 2.3 Total suspended solid (TSS)

Surface water samples were collected using 500 ml brown Nalgene bottles. Bottom water samples, i.e., 20 cm above sediment, were collected using Van Dorn sampler. Two replicate water samples were taken at each station. Water samples were kept on ice after collection and filtered within 24 hours of collection using GF/F filter paper. Filtered sediment samples were dried and weighed to determine TSS.

### 2.4 Chlorophyll-α and inorganic nutrients

Water samples were collected from surface water and bottom water for chlorophyll-α, filtered (GF/F filter paper) on site and then stored frozen. Chlorophyll-α was determined using non-acidic extraction method. A Turner Design Trilogy Fluorometer (Model# 7200) was used to measure chlorophyll-α concentration using a methanol extract method [7]. Analysis was performed within 12–16 h of methanol addition.

Nutrient samples were filtered on site using 0.45 μm polycarbonate filter paper and kept on ice until stored frozen, and were processed for analysis within two weeks. Inorganic nutrients data were obtained using an OIA segmented flow auto-analyzer (Xylem Brand, Rye Brook, NY, USA that combines both segmented flow analysis and flow injection analysis techniques with computer controlled sample selection and peak processing.

Nutrient chemistries were measured as specified by the manufacturer of OIA segmented flow auto-analyzer. The range of method detection limits (MDL) are 0.1–10 μmol/L for ammonium (NH_4_^+^), 0.02–10 μmol/L for orthophosphate (o-PO_4_), 0.35–35 μmol/L for silica (SiO_2_), and 0.02–40 μmol/L for nitrite + nitrate (NO_3_^−^ + NO_2_^−^, here also referred as NO_x_). Silica in samples reacts with molybdate in acid medium and is detected as silicic acid or silicate. Matrix matching between the carrier, standards and the sample matrix minimizes refractive index effects on absorbance, which are caused in part by salinity. Artificial seawater is adequate for the analysis of both o-PO_4_ and SiO_2_, but matrix matching is important for dissolved nitrogen (N) chemistries and requires the use of low nutrient seawater (LNSW) to accurately detect low (μmol) levels of N in samples. The typical lowest concentration minimum reportable levels (LCMRL) are: 0.25–10.0 μmol NO_x_ (O.I. Analytical method 15040908, OIA 2008), 10.0–300.0 μmol SiO_2_ (O.I. Analytical method 15061001, OAI 2001a), and 0.25–10.0 μmol NH_4_^+^ (O.I. Analytical method 15031107, OIA, 2007). The o-PO_4_ method has a LCMRL of 0.10–10.0 μmol (Perstorp Analytical method 000589 OIA 2001b), but is a modification of the Alpkem chemistries method [8]. In the present study LCMRL was used to prepare standard curve for the analyses.
