# Supplementary material for: Water quality data from estuarine variable hydrologic flow regimes during frequent drought
Source: Data Brief. 2019 Jun 26;25:104178. doi: 10.1016/j.dib.2019.104178 (PMC6612631; doi:10.1016/j.dib.2019.104178)
Supplement: Supplementary file 1 [file mmc1.docx]

libname bhanu 'N:\Bhanu\TSS_nut paper';

**data** nut;

attrib TRT length=$**4**;

attrib Treatment length=$**4**;

set Bhanu.allbaytssnut;

Treatment=scan(Place,**1**);

TRT=compress(Est || "-" || substr(place,**1**,**1**));

**run**;

**proc** **sort** data=nut;

by EST Date Sta Depth;

**run**;

**To calculate weighted means**

**proc** **means** data=nut noprint;

by EST Date Sta depth;

id Treatment Trt;

var Temp Sal DO pH Secchi PO4 SiO4 NH4 NOx Chl TSS POM ; *AFDW POM missing;

output out=depth mean=Temp Sal DO pH Secchi PO4 SiO4 NH4 NOx Chl TSS POM;

**run**;

**proc** **means** data=depth noprint;

by EST Date Sta;

id Treatment trt;

var Temp Sal DO pH Secchi PO4 SiO4 NH4 NOx Chl TSS POM;

output out=sta mean=Temp Sal DO pH Secchi PO4 SiO4 NH4 NOx Chl TSS POM;

**run**;

**proc** **sort** data=sta;

by Est Date Treatment Sta;

**run**;

**proc** **means** data=sta noprint;

by EST Date Treatment; id Trt;

var Temp Sal DO pH Secchi PO4 SiO4 NH4 NOx Chl TSS POM;

output out=nutt(drop=_type_ _freq_) mean=Temp Sal DO pH Secchi PO4 SiO4 NH4 NOx Chl TSS POM;

**run**;

**proc** **sort** data=nutt; by Trt date; **run**;

**To obtain Fig 2 of Paudel et al. 2019 paper**

title;

%***modstyle*** (parent=journal, name=NFWF, TYPE=CLM, markers=circle circlefilled triangle trianglefilled square squarefilled);

ods html dpi=**300** style=NFWF sge=on gpath='C:\Users\pmontagna\Documents\Students\Paudel\Nutr Paper';

ods graphics on / border=off width=**3**in height=**6**in imagename='Mixing';

**proc** **sgscatter** data=nutt;

compare x=Sal y=(NH4 NOx PO4 SiO4) /group=trt ;

label TRT='Estuary-Treatment' Sal='Salinity (psu)'

NH4='NH4 (µmol/L)' NOx='NOx (µmol/L)' PO4='PO4 (µmol/L)' SiO4='SiO4 (µmol/L)';

**run**;**quit**;

**proc** **sort** data=nutt; by Est date treatment; **run**;

**proc** **means** data=nutt noprint;

by EST Date;

var Temp Sal DO pH Secchi PO4 SiO4 NH4 NOx Chl TSS POM;

output out=nuttime(drop=_type_ _freq_) mean=Temp Sal DO pH Secchi PO4 SiO4 NH4 NOx Chl TSS POM;

**run**;
